# Impaired AMPA signaling and cytoskeletal alterations induce early synaptic dysfunction in a mouse model of Alzheimer's disease

**DOI:** 10.1111/acel.12791

**Published:** 2018-06-06

**Authors:** David Baglietto‐Vargas, Gilberto Aleph Prieto, Agenor Limon, Stefania Forner, Carlos J. Rodriguez‐Ortiz, Kenji Ikemura, Rahasson R. Ager, Rodrigo Medeiros, Laura Trujillo‐Estrada, Alessandra C. Martini, Masashi Kitazawa, Jose C. Davila, Carl W. Cotman, Antonia Gutierrez, Frank M. LaFerla

**Affiliations:** ^1^ Institute for Memory Impairments and Neurological Disorders University of California Irvine California; ^2^ Department of Neurobiology and Behavior University of California Irvine California; ^3^ Department of Cell Biology, Genetic and Physiology Faculty of Sciences Biomedical Research Institute of Malaga (IBIMA) Networking Research Center on Neurodegenerative Diseases (CIBERNED) University of Malaga Malaga Spain; ^4^ Department of Psychiatry and Human Behavior University of California Irvine California; ^5^ Division of Occupational and Environmental Medicine Department of Medicine Center for Occupational and Environmental Health (COEH) University of California Irvine California; ^6^ Clem Jones Centre for Ageing Dementia Research Queensland Brain Institute The University of Queensland Brisbane Qld Australia; ^7^ Department of Neurology University of California Irvine California

**Keywords:** actin cytoskeleton, Alzheimer's disease, AMPA receptor, Aβ, immunotherapy, synaptic impairment

## Abstract

Alzheimer's disease (AD) is a devastating neurodegenerative disorder that impairs memory and causes cognitive and psychiatric deficits. New evidences indicate that AD is conceptualized as a disease of synaptic failure, although the molecular and cellular mechanisms underlying these defects remain to be elucidated. Determining the timing and nature of the early synaptic deficits is critical for understanding the progression of the disease and for identifying effective targets for therapeutic intervention. Using single‐synapse functional and morphological analyses, we find that AMPA signaling, which mediates fast glutamatergic synaptic transmission in the central nervous system (CNS), is compromised early in the disease course in an AD mouse model. The decline in AMPA signaling is associated with changes in actin cytoskeleton integrity, which alters the number and the structure of dendritic spines. AMPA dysfunction and spine alteration correlate with the presence of soluble but not insoluble Aβ and tau species. In particular, we demonstrate that these synaptic impairments can be mitigated by Aβ immunotherapy. Together, our data suggest that alterations in AMPA signaling and cytoskeletal processes occur early in AD. Most important, these deficits are prevented by Aβ immunotherapy, suggesting that existing therapies, if administered earlier, could confer functional benefits.

## 1 INTRODUCTION

The pathobiology of Alzheimer's disease (AD) is complex and involves changes in many different cellular process, such as alterations in the blood–brain barrier (BBB), synaptic function, metabolic process, oxidative stress, and inflammation (Forner, Baglietto‐Vargas, Martini, Trujillo‐Estrada & LaFerla, 2017). Among these pathological processes, new evidence from epidemiological studies suggests that synaptic loss is the best predictor of the clinical symptoms of patients with AD (Penzes, Cahill, Jones, VanLeeuwen & Woolfrey, 2011; Selkoe, 2002; Walsh & Selkoe, 2004). Therefore, identifying the mechanisms by which these deficits occur continues to be an intense area of investigation. A major ongoing question in the field seeks to better understand the molecular and cellular mechanisms that take place during the early stages of the disease that leads to the development of cognitive impairments. This is a critical question to address, as new clinical trials have started to treat patients when memory loss is mild or absent, with the goal of stopping further synaptic damage before it becomes irreversible (McDade & Bateman, 2017). Thus, it is vital to elucidate the earliest possible neuronal changes relevant to the development of cognitive deficits so that more effective treatments can be developed.

Amino‐3‐hydroxy‐5‐methyl‐4‐isoxazolepropionic acid receptors (AMPARs) play a central role in the modulation of excitatory synaptic transmission in the central nervous system (CNS) (Guntupalli, Widagdo & Anggono, 2016; Hanley, 2008; Hsieh et al., 2006). AMPARs are composed of four different subunits (glutamate receptor subunits A1 to A4), which regulate distinct functional aspects of the receptors (Hanley, 2008; Shepherd & Huganir, 2007). For example, subunits with long intracellular carboxy termini are involved in activity‐dependent synaptic targeting of AMPARs (GluA1, GluA2L, and GluA4), whereas those with a short intracellular carboxy end maintain basal synaptic neurotransmission (GluA2, GluA3, and GluA4s) (Hanley, 2008; Shepherd & Huganir, 2007). The role of the GluA1 subunit in learning and memory processes has been the most extensively investigated (Guntupalli et al., 2016; Hanley, 2008; Hsieh et al., 2006). The GluA1 subunit has several phosphorylation sites on its intracellular carboxy terminus (including Ser818, Ser831, and Ser845), and many of these sites are involved in the regulation of AMPARs at the synapse (Olivito et al., 2016). For example, phosphorylation at Ser831 and Ser845 regulates the conductivity of the ion channel and the translocation of the AMPAR to the surface membrane, respectively (Olivito et al., 2016). In an interesting manner, recent studies revealed that GluA1‐dependent synaptic transmission is impaired in AD, although its involvement in memory and cognitive process in AD remains unknown (Hsieh et al., 2006).

One of the most significant structural changes that occur during learning, memory, and other cognitive processes is the restructure of dendritic spines (Parajuli, Tanaka & Okabe, 2016). Given their important role in neuronal plasticity, defects specific to dendritic spines are likely to play a critical role in the synaptic and cognitive impairments associated with AD. Consistent with this idea, numerous pre‐ and postsynaptic proteins have been shown to be perturbed in AD patients, suggesting that spine defects play a central role in cognitive decline in AD (Masliah et al., 2001). However, the exact mechanism(s) underlying these changes are poorly understood. Hence, elucidating the mechanisms by which dendritic spines and synaptic function are affected in AD, particularly in the prodromal stage, will provide greater insight into the onset of cognitive deficits in patients with AD and potentially lead to the identification of novel biomarkers and treatment targets.

Here, we used the preclinical 3xTg‐AD transgenic mouse model to determine the mechanism by which synaptic function and dendritic spines are affected early in the progression of the disease. Previous studies from our laboratory have shown that 3xTg‐AD mice develop earlier cognitive impairments, although the mechanism underlying the deficits remained unknown (Oddo et al., 2003). This study concludes that ultrastructural changes in the dendritic spines, and functional AMPA signaling alterations, lead to robust synaptic impairments. These structural synaptic alterations were associated with changes in several key factors (such as drebrin and spinophilin) that regulate the actin cytoskeleton. In an important way, Aβ immunotherapy reversed the synaptic defects observed in the 3xTg‐AD mice, suggesting that an earlier intervention is a promising therapeutic approach.

## 2 RESULTS

### 2.1 Synaptic function is impaired early in the 3xTg‐AD hippocampus

**Figure 1:**
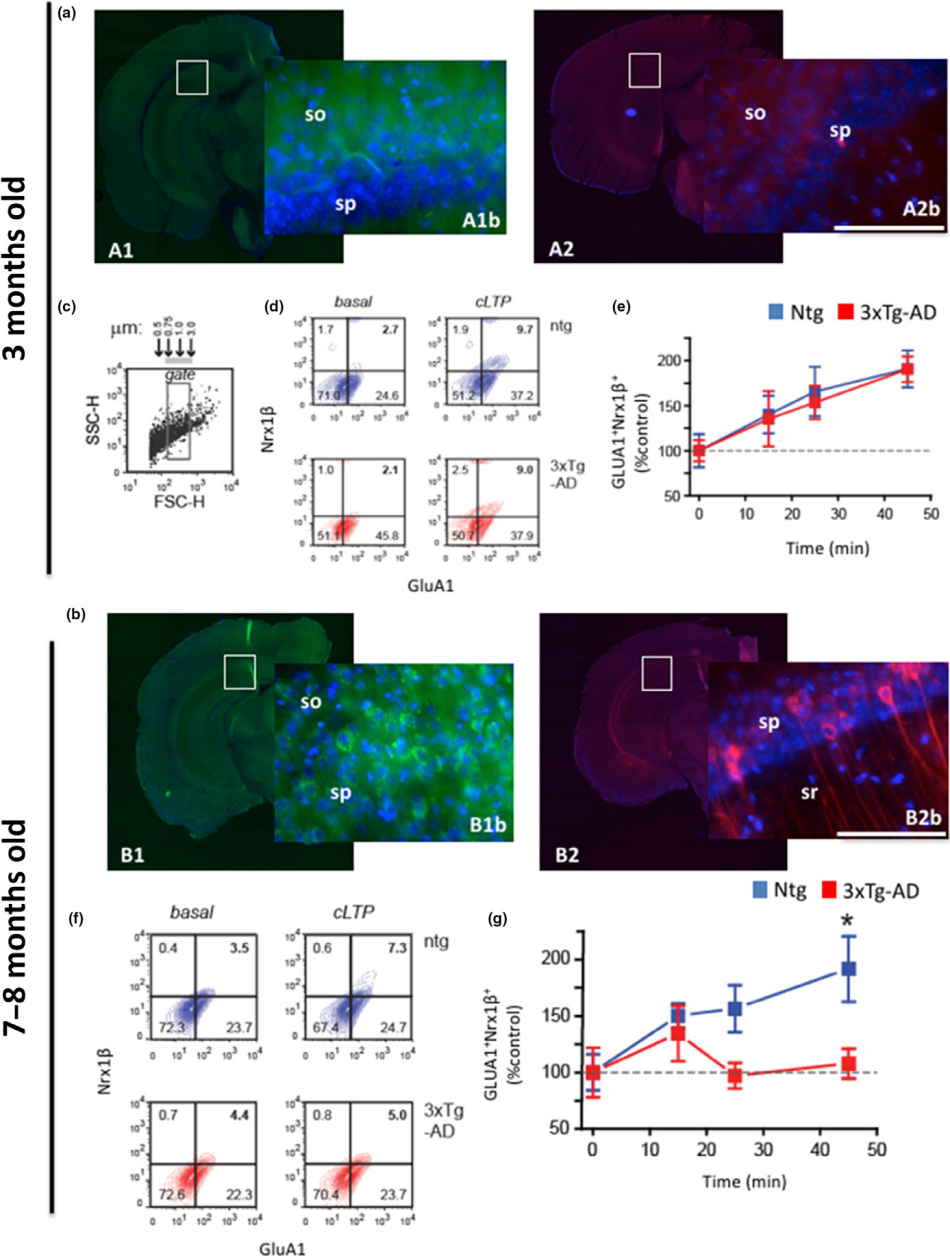
Synaptic function is early affected in 3xTg‐AD mice. (a) Confocal images stained with 6E10 (for Aβ, A1) and HT7 (for tau, A2) in 3‐month‐old 3xTg‐AD mice show no immunoreactivity at this age. (b) Confocal images stained with 6E10 (for Aβ) and HT7 (for tau) with Dapi in 7‐ to 8‐month‐old 3xTg‐AD mice show intracellular immunostain for both antibodies in the CA1 hippocampus. (c–e) Chemical long‐term potentiation (cLTP) in synaptosomes from Ntg and 3xTg‐AD mice at 3 months of age revealed no differences in the percentage of GluA1^+^‐Nrx1β^+^ events at 0, 15, 25, and 45 min after cLTP induction. (f,g) cLTP in synaptosomes from Ntg and 3xTg‐AD mice at 7–8 months of age showed significant differences in the percentage of GluA1^+^‐Nrx1β^+^ events at 45 min after cLTP induction (191.8 ± 28.9 (Ntg) vs. 107.8 ± 13.2 (3xTg‐AD), *p <* *0.05, *t* test). The basal levels of synaptosomes were similar in both Ntg and 3xTg‐AD mice. The values represent the mean ± *SEM* (*n* = 5–7 per group). *p <* *0.05. Scale bars: 100 μm (A1b, A2b, B1b, and B2b). so: stratum oriens, sp: stratum pyramidale, sr: stratum radiatum

We first determined whether synaptic function was impaired in 3xTg‐AD mice at a time point when only soluble Aβ or tau was present (Oddo et al., 2003). We initially selected an age with no sign of pathology (3 months; Figure 1a) and another when amyloid or tau pathology is just emerging (7–8 months; Figure 1b). Synaptic plasticity in hippocampal synaptosomes was assessed by fluorescence analysis of single‐synapse long‐term potentiation (FASS‐LTP) (Prieto et al., 2015). This flow cytometry‐based method induces chemical LTP in freshly isolated synaptosomes by chemical stimulation and tracks the insertion of glutamate AMPARs (GluA1) in the postsynaptic surface and Neurexin1β (Nrx1β) in the presynaptic surface, to ensure that intact synaptosomes contain both presynaptic and postsynaptic elements (Prieto et al., 2015). FASS‐LTP identified synaptosomes by size using calibrated beads (0.5–3.0 μm) during flow cytometry analysis (Figure 1c). LTP was induced in Ntg and 3xTg‐AD hippocampal synaptosomes by chemical stimulation (45 min, glycine‐KCl stimulation), and potentiated synapses were identified by selecting double‐labeled GluA1^+^‐Nrx1β^+^ synaptosomes (Figure 1d). At the beginning, we selected an age with no sign of pathology (3 months; Figure 1a) and no differences between Ntg and 3xTg‐AD mice were observed at this age (Figure 1d,e). Furthermore, no evidence of synaptic alterations was detected in Ntg or 3xTg‐AD hippocampus at 3 months of age (Figure  S2A,B). In contrast, FASS‐LTP analysis revealed that the percentage of GluA1^+^‐Nrx1β^+^ events induced by cLTP was significantly reduced in the 3xTg‐AD compared to Ntg mice at 7–8 months of age (Figure 1f,g), correlating with the emergence of Aβ and tau pathology (Figure 1b), indicating that 3xTg‐AD mice exhibit a significant decrease in synaptic cLTP responses (Figure 1g). These data are consistent with a previous study from our laboratory that showed that electrically induced LTP is impaired in hippocampal slices of 6‐month‐old 3xTg‐AD mice (Oddo et al., 2003). Overall, these findings suggest that 3xTg‐AD mice display significant earlier synaptic impairment when only soluble Aβ or tau is present.

### 2.2 Synaptosomal AMPAR ion currents are compromised in 3xTg‐AD mice

FASS‐LTP measurements showed that the insertion of hippocampal GluA1‐containing AMPARs during cLTP was significantly reduced in 3xTg‐AD mice. To determine whether functional impairment of synaptic AMPARs was also observed at this age, we analyzed the electrophysiological responses of AMPARs from Ntg and 3xTg‐AD hippocampal synaptosomal membranes microtransplanted to *Xenopus* oocytes using microtransplanted synaptic membranes (MSMs) and two‐electrode voltage clamp procedures (Limon, Reyes‐Ruiz & Miledi, 2012). MSMs allow the electrophysiological analysis of the same samples used in FASS‐LTP and western blots. Moreover, microtransplanted receptors are recorded while still embedded in their original lipid milieu and complexed with their associated proteins (Limon et al., 2012).

**Figure 2:**
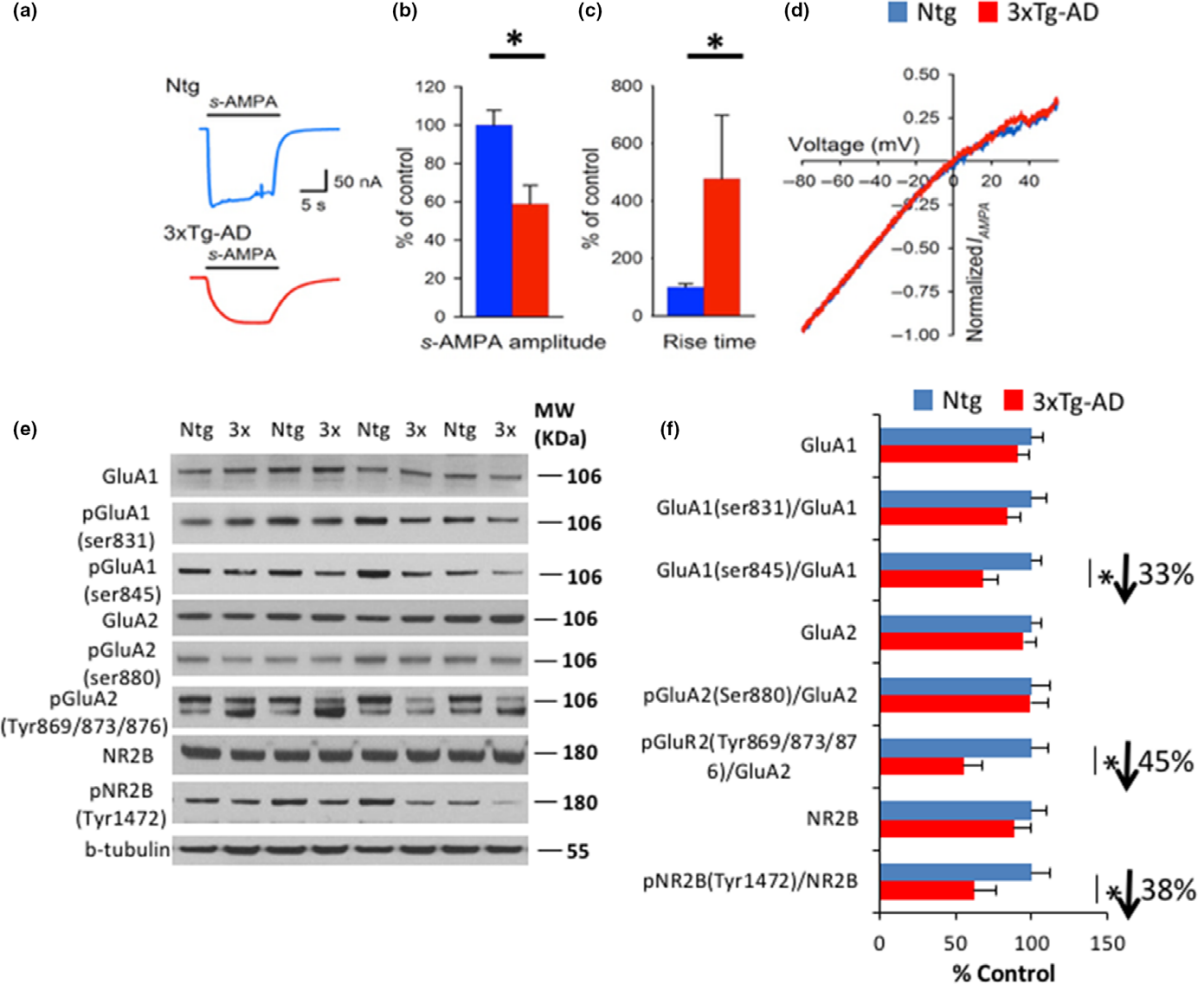
Early functional alterations of AMPA receptors in 3xTg‐AD mice. (a) and (b) Electrophysiological recordings of synaptic‐enriched membrane preparations by microtransplantation of synaptic membranes (MSM) showed a 41% reduction in ion currents through α‐amino‐3‐hydroxy‐5‐methyl‐4‐isoxazolepropionic acid receptors (AMPAR) in 3xTg‐AD samples (*p <* *0.05, *t* test, *n* = 5 Ntg mice, *n* = 4 3xTg‐AD mice). AMPARs were stimulated with 10 μM *s*‐AMPA coapplied with 10 μM cyclothiazide (CTZ) after preincubation in 10 μM CTZ for 3 min to remove AMPAR desensitization. (c) AMPARs from 3xTg‐AD mice showed a highly variable rise time of activation, defined as the time from the current onset to the maximum amplitude that was 4.5‐fold slower compared to Ntg (*p <* *0.05, *t* test). The figure shows the slowest current observed in 3xTg‐AD mice. D) No difference was observed in the current–voltage relationship of steady‐state ion currents elicited with voltage ramps from −80 to +50 mV. E‐F) Immunoblot analysis of GluA1, GluA2, pGluA1, pGluA2, NR2B, and pNNR2B from hippocampal synaptosome homogenates of 7‐ to 8‐month‐old Ntg and 3xTg‐AD mice is shown as alternating lanes. Quantification of pGluA1, pGluA2, and pNR2B normalized to GluA1, GluA2, and NR2B and expressed as a % of control shows significant decreases in pGluA1 (32.22 ± 10.74, *p <* *0.05, *t* test), pGluA2 (44.86 ± 12.76, *p <* *0.05, *t* test), and pNR2B (37.84 ± 14.05, *p <* *0.05, *t* test) between 3xTg‐AD mice and Ntg mice. The values represent the mean ± *SEM* (*n* = 4–7 per group). *p <* *0.05

AMPAR responses were measured after 180‐s preincubation in 10 μM cyclothiazide (CTZ) to remove the desensitization of AMPARs and measure the maximum response amplitude (Limon et al., 2012). This analysis showed that AMPA+CTZ‐elicited currents in oocytes injected with 3xTg‐AD membranes were significantly smaller compared to those injected with Ntg membranes (Figure 2a,b). In particular, kinetic alterations were also observed in 7‐ to 8‐month‐old 3xTg‐AD mice. Activation of AMPA‐elicited currents was significantly slower in synaptic membranes from 3xTg‐AD mice relative to Ntg controls (Figure 2a,c). No alterations in the voltage‐dependent rectification index of AMPARs were observed, indicating that no major changes in the proportion of AMPAR subunits were present in the microtransplanted receptors (Washburn, Numberger, Zhang & Dingledine, 1997) (Figure 2d). These data suggest that functional alterations in synaptic AMPARs are present at early stages in 3xTg‐AD model development.

Western blot analysis revealed significant decreases in the phosphorylation state of multiple AMPAR and NMDAR subunits. These findings are significant because post‐translational modifications regulate the incorporation of AMPARs into the synapses and also facilitate the internalization of NMDARs (Snyder et al., 2005) (Figure 2e,f). Therefore, the post‐translational modifications of AMPARs and NMDARs may be associated with the synaptic deficits observed in 3xTg‐AD mice.

Together, these findings suggest that AMPAergic synaptic function is affected in 3xTg‐AD mice well before the onset of overt plaque and tangle formation.

### 2.3 3xTg‐AD mice exhibit important dendritic spine alterations

**Figure 3:**
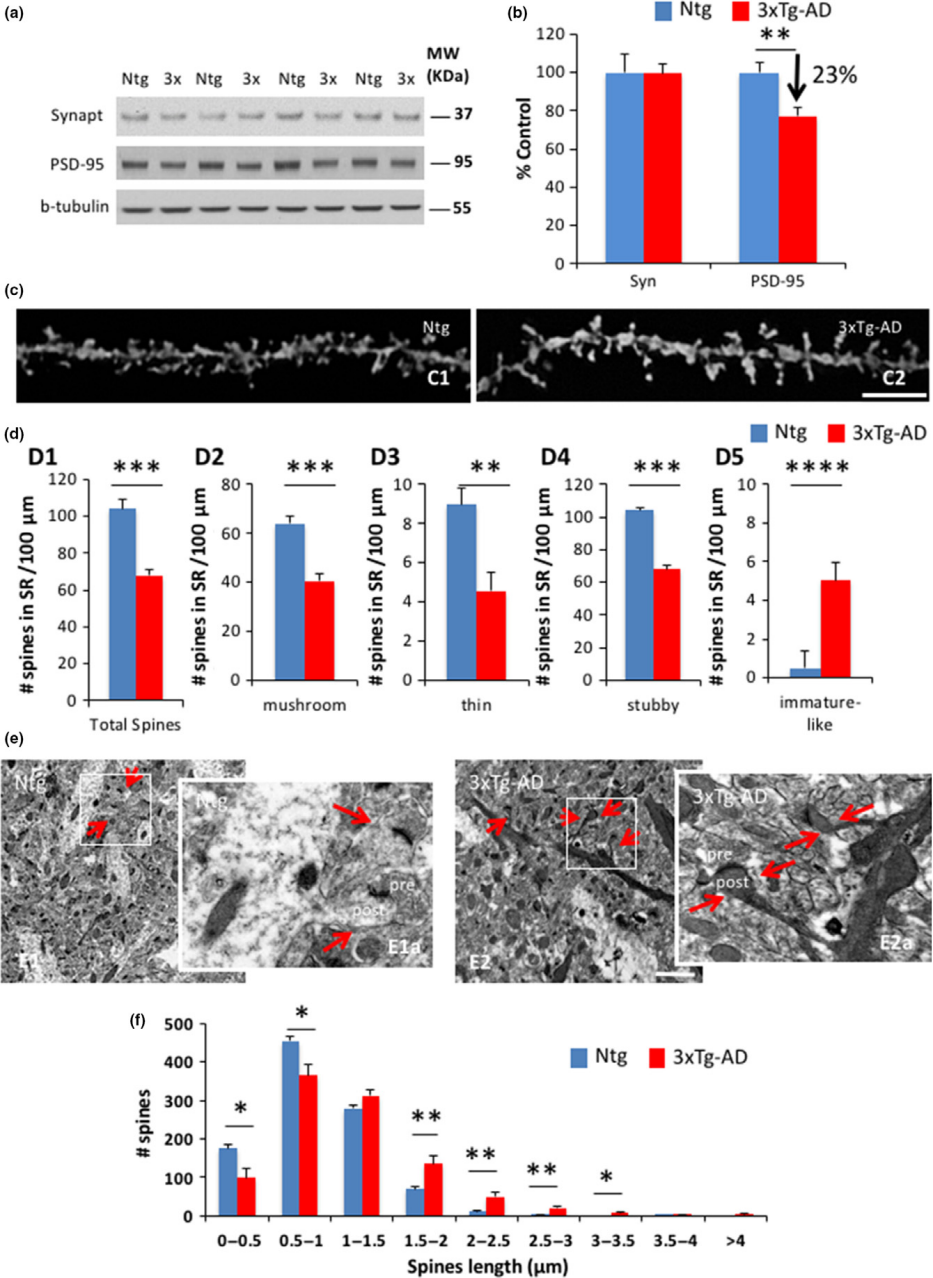
3xTg‐AD mice display morphological and structural dendritic spine alterations. (a,b). Immunoblot analysis of synaptophysin (Synapt) and postsynaptic density protein 95 (PSD‐95) from hippocampal synaptosome homogenates of 7‐ to 8‐month‐old Ntg and 3xTg‐AD mice is shown as alternating lanes. Quantification of PSD‐95 normalized to b‐tubulin and expressed as a % of control shows significant differences between Ntg and 3xTg‐AD mice (−22.93 ± 4.43, *p <* *0.05, *t* test). (c,d) Light microscopic 3D reconstruction images of radiatum layer in CA1 subfield in Ntg (C1) and 3xTg‐AD (C2) mice. (d) Stereological quantification showed a significant decrease in total (34.62 ± 2.84, ***p <* *0.001, *t* test), mushroom (36.67 ± 4.59, ***p <* *0.001, *t* test), and thin (48.91 ± 9.47, **p <* *0.01, *t* test) and stubby (41.47 ± 6.06, ***p <* *0.001, *t* test) spines. In particular, immature‐like spines were increased in 3xTg‐AD mice compared to Ntg mice (80.60 ± 3.89, ****p <* *0.0001, *t* test). (e) Electron microscopic images showed the appearance of electrodense dendritic spines in 3xTg‐AD compared to Ntg mice. (d) Spine length is increased in 3xTg‐AD compared to Ntg mice. The values represent the mean ± *SEM* (*n* = 5–7 per group). *p < 0.05, **p < 0.01, ***p < 0.001, and ****p < 0.001. Scale bars: 5 μm (C1 and C2) and 2 μm (E1 and E2)

Dendritic spines are specialized postsynaptic structures that cover neuronal processes. The earlier synaptic impairments described in the 3xTg‐AD mice could be associated with structural and/or morphological alterations of the dendritic spines. In an important way, deficits in the postsynaptic density protein 95 (PSD‐95) were observed in hippocampal synaptosomes in 3xTg‐AD compared to Ntg mice, supporting the idea that alterations in postsynaptic structures may occur in 3xTg‐AD mice (Figure 3a,b). To investigate this further, Golgi staining and stereological analysis in the stratum radiatum of the CA1 hippocampus were performed in 7‐ to 8‐month‐old Ntg and 3xTg‐AD mice. The quantification of dendritic spines indicated that 3xTg‐AD mice displayed significant deficits in spine density compared to Ntg mice, including mushroom and thin and stubby spines (Figure 3c,d). In an interesting manner, 3xTg‐AD mice displayed an elevated number of longer and/or immature‐like spines compared to Ntg mice (Figure 3d). Electron microscopic images confirmed that 3xTg‐AD mice displayed more electrodense, longer, and immature‐like spines compared to Ntg animals (Figure 3e). Furthermore, stereological quantification corroborated that Ntg mice have shorter spines, whereas 3xTg‐AD mice have an increased number of longer spines (Figure 3f). The immature‐type and electrodense dendritic spines were particularly evident in pyknotic pyramidal neurons in 3xTg‐AD mice (Figure S3A). In addition, dendritic width was diminished in these mice (Figure S3B,C), and Sholl analysis showed no differences in the number of dendritic intersections in 3xTg‐AD mice compared to Ntg mice (Figure S3D,E). Overall, our data indicate that 3xTg‐AD mice exhibit profound morphological and structural synaptic changes that correlate with the impairments in synaptic function in this transgenic model.

### 2.4 Actin‐binding proteins are diminished in 3xTg‐AD mice

**Figure 4:**
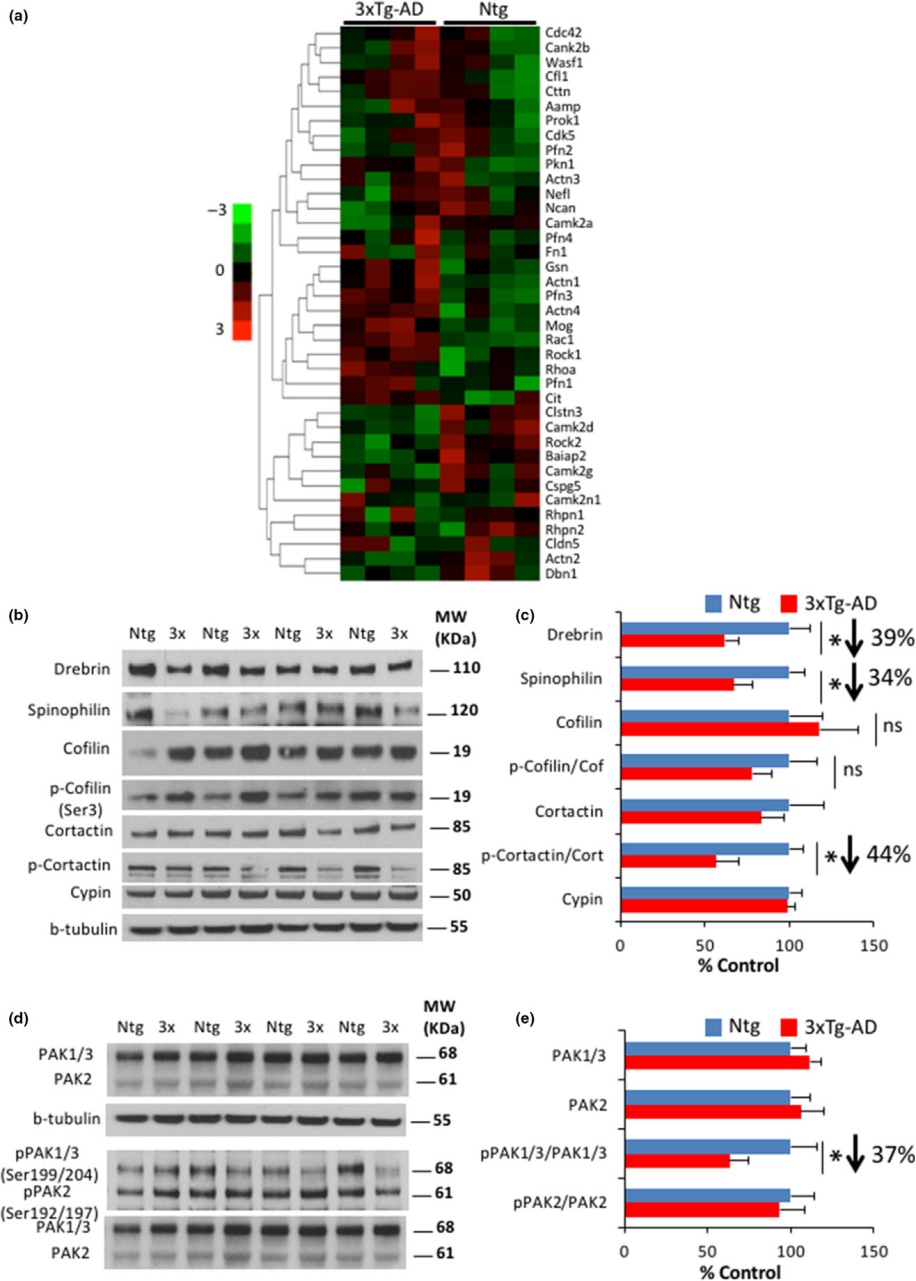
Actin‐binding regulators are extensively affected in 3xTg‐AD mice. (a) NanoString technology was employed to assess RNA transcript levels of 37 cell adhesion and cytoskeleton synaptic genes (*n* = 4 per group). Green indicates downregulation of gene expression, and meanwhile, red indicates upregulation. The actin‐binding RNA heat‐map showed important changes in Ntg vs. 3xTg‐AD mice. (b,c) Immunoblot analysis of drebrin, spinophilin, cofilin, p‐cofilin, cortactin, p‐cortactin, and cypin from hippocampal synaptosome homogenates of 7‐ to 8‐month‐old Ntg and 3xTg‐AD mice is shown as alternating lanes. Quantification of drebrin, spinophilin, and p‐cortactin normalized to b‐tubulin and cortactin, respectively, and expressed as a % of control revealed significant decreases in drebrin (38.39 ± 8.04, *p <* *0.05, *t* test), spinophilin (33.03 ± 11.36, *p <* *0.05, *t* test), and p‐cortactin (43.80 ± 14.01, *p <* *0.05, *t* test) in 3xTg‐AD mice compared to Ntg mice. (d) Immunoblot analysis of PAK and phosphor‐PAK from hippocampal synaptosome homogenates of 7‐ to 8‐month‐old Ntg and 3xTg‐AD mice is shown as alternating lanes. (e) Quantification of PAK1‐3 normalized to b‐tubulin and expressed as a % of control showed no differences between Ntg and 3xTg‐AD mice. However, phospho‐PAK1/3 (36.64 ± 6.95, *p <* *0.05, *t* test) was significantly decreased in 3xTg‐AD compared to Ntg mice. The values represent the mean ± *SEM* (*n* = 4–7 per group). *p < 0.05, **p < 0.01, and ***p < 0.001. Scale bar: 5 μm (C1 and C2), 2 μm (E1 and E2)

We next investigated the underlying molecular mechanism responsible for the significant structural dendritic alterations found in 3xTg‐AD mice. The structure and function of dendritic spines are dynamically regulated by the actin cytoskeleton (Cingolani & Goda, 2008; Hotulainen & Hoogenraad, 2010), and we observed numerous changes in the mRNA expression of multiple key regulators of actin cytoskeleton dynamics (*Act1, Actn2, Actn3, Actn4, Dbn1, Cfl1, Camk2a, Camk2b, Camk2 g, Camk2n1, Rac1, Cdc42, Rhoa, Rock1, Rock2, Pfn1, Pfn2, Pfn3, Pfn4, Cit, Pkn1, Prok1, Rhpn1, Rhpn2, Gsn, Cttn, Wasf1, Cdk5, Baiap2, Cldn5, Nefl, Fn1, Aamp, Cspg5, Clstn3, Mog,* and *Ncan*), including upregulation or downregulation, in the hippocampi of 7‐month‐old 3xTg‐AD mice compared to Ntg animals (Figures 4a and S4A). Furthermore, an important intragroup variability arising from the tissue samples was also observed for some of the expressed genes (e.g., top and bottom clusters in Figure 4a). However, the expression of genes that showed statistical significance (middle cluster) shows minimal intragroup variance (Figure 4a). The intragroup variability at the top and bottom clusters could be due to a less consistent pathology in male 3xTg‐AD mice (Carroll et al., 2010). To correlate the RNA changes at the protein level, western blot analysis demonstrated that the levels of multiple actin‐binding proteins (including drebrin, spinophilin, and p‐cortactin) were significantly reduced in the 3xTg‐AD mice (Figure 4c,d). In addition, the activation of p21‐activated kinase (PAK) was also diminished in 3xTg‐AD mice (as evidenced by a reduction in the phosphorylated form (Sells, Pfaff & Chernoff, 2000)) (Figure 4e,f). These data suggest that impairments in the actin cytoskeleton are responsible for the dendritic morphological changes observed in 3xTg‐AD mice.

### 2.5 Aβ and phospho‐tau accumulated in the synaptic compartment of 3xTg‐AD mice

**Figure 5:**
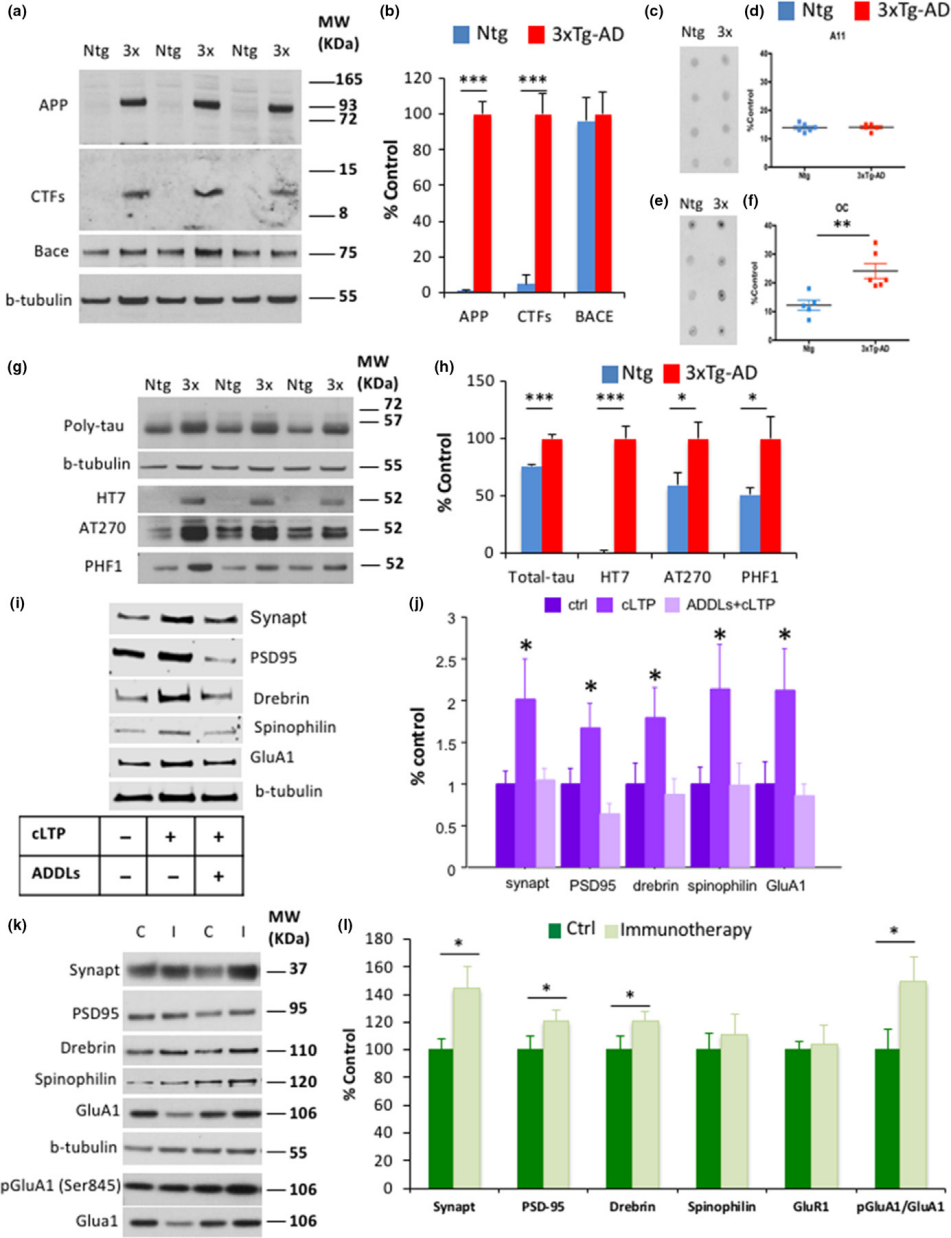
Aβ pathology induces synaptic loss in 3xTg‐AD mice. (a,b) Immunoblot analysis of amyloid precursor protein (APP), C‐terminal fragments (CTFs), and β‐secretase 1 (BACE1) from hippocampal synaptosome homogenates of 7‐ to 8‐month‐old Ntg and 3xTg‐AD mice is shown as alternating lanes. Quantification of APP and CTFs normalized to b‐tubulin and expressed as a % of control indicated significant increases in APP (97.97 ± 1.67, ***p <* *0.001, *t* test) and CTFs (95.97 ± 2.48, ***p <* *0.001, *t* test) in 3xTg‐AD mice compared to Ntg mice. (c–f) Dot blot analysis showed a significant increase in the level of Aβ‐oligomers recognized by the antibody OC (69.53 ± 18.42, ***p <* *0.01, *t* test) in 3xTg‐AD compared to Ntg mice. (g,h) Immunoblot analysis of total tau (poly‐tau), human tau (HT7), phospho‐tau Thr181 (AT270), and Ser396/Ser404 (PHF1) from hippocampal synaptosome homogenates of 7‐ to 8‐month‐old Ntg and 3xTg‐AD mice is shown as alternating lanes. Quantification of total tau, HT7, AT270, and PHF1 normalized to b‐tubulin and expressed as a % of control showed significant increases in total tau (24.30 ± 2.09, ***p <* *0.001, *t* test), HT7 (99.99 ± 16.25, ***p <* *0.001, *t* test), AT270 (40.51 ± 29.50, *p <* *0.05, *t* test), and PHF1 (49.22 ± 37.35, *p <* *0.05, *t* test) in 3xTg‐AD mice compared to Ntg mice. (i) Immunoblot analysis of synaptic markers, actin‐binding proteins, and total GluA1 protein levels in hippocampal primary cell cultures after ADDL incubation and cLTP induction. (j) Quantification of A. The values represent the mean ± *SEM* (*N* = 8–9 per group). (k,l) Immunoblot analysis of synaptophysin, PSD‐95, drebrin, spinophilin, GluA1, and pGluA1 from hippocampal synaptosome homogenates of 7‐month‐old 3xTg‐AD‐control and 3xTg‐AD‐6E10 mice is shown as alternating lanes. Quantification synaptophysin, PSD‐95, drebrin, spinophilin, GluA1, and pGluA1 (ser845) normalized to b‐tubulin or GluA1, respectively, and expressed as a % of control revealed a significant increase in synaptophysin (44.22 ± 16.72, *p <* *0.05, *t* test), PSD‐95 (20.87 ± 8.12, *p <* *0.05, *t* test), drebrin (49.17 ± 14.61, *p <* *0.05, *t* test), and pGluA1/GluA1 levels (24.38 ± 8.72, *p *<* *0.07, *t* test), in 3xTg‐AD‐6E10 mice compared to 3xTg‐AD‐control mice. The values represent the mean ± *SEM* (*N* = 4–7 per group). *p <* *0.05, **p <* *0.01, and ***p <* *0.001

We next investigated the nature and amount of AD pathology (including Aβ and tau pathology) in synapses. Western blot analysis in hippocampal synaptosomes showed that the human amyloid precursor protein (APP) and C‐terminal fragments (CTFs) (recognized both by the 6E10 antibody) were significantly elevated in 3xTg‐AD mice, with no expression observed in Ntg mice (Figure 5a,b). Moreover, the steady‐state levels of β‐secretase 1 (BACE1) remained unchanged in the 3xTg‐AD and Ntg mice (Figure 5a,b). Dot blot analysis showed that fibrillar oligomers (recognized by the OC antibody) were elevated in the 3xTg‐AD vs. Ntg mice, whereas soluble oligomers (recognized by the A11 antibody) remained unchanged (Figure 5c–f). We further evaluated tau pathology in Ntg and 3xTg‐AD synaptosomes, and western blot analysis showed significant accumulation of total tau in 3xTg‐AD mice compared to Ntg mice, due in part to the presence of both endogenous and human tau in 3xTg‐AD mice (Baglietto‐Vargas et al., 2014) (Figure 5g,h). Western blot analysis also revealed a significant increase in tau phosphorylated at residues Thr181 (recognized by the AT270 antibody) and Ser396/404 (recognized by the PHF1 antibody) in the 3xTg‐AD compared to Ntg mice. These data suggest that both synaptic Aβ and tau might affect the synaptic morphology and function in 3xTg‐AD mice. In addition, significant increases in the human APP, soluble and insoluble oligomers, and tau pathology were detected in the soluble protein fraction (S1) in 3xTg‐AD mice (Figure S5A–E). Therefore, it is possible that extrasynaptic Aβ and tau pathology could also contribute to the disruption of the synaptic function in the 3xTg‐AD mice.

### 2.6 Aβ immunotherapy mitigates early synaptic deficits in 3xTg‐AD mice

We next sought to determine whether the synaptic changes observed in the hippocampus of 3xTg‐AD mice are associated with the Aβ accumulation that this mouse model presents. First, we used a cell culture approach to determine whether Aβ oligomers are capable of altering the levels of plasticity‐related proteins. We observed increased protein levels in the pre‐ and postsynaptic markers synaptophysin and PSD‐95 1 h after cLTP stimulation in primary hippocampal cells (Figure 5i,j). In the same way, the total GluA1 level was augmented after cLTP induction, and we also observed increased expression of the actin‐binding proteins drebrin and spinophilin (Figure 5i,j). In an important way, Aβ oligomers (ADDLs) induced significant reductions in the synaptic markers, actin‐binding proteins, and total GluA1 protein levels observed after cLTP stimulation (Figure 5i,j). These results suggest that ADDLs induce profound impairments in cLTP and dysregulate synaptic, cytoskeleton‐binding, and AMPAR proteins.

Furthermore, a passive intracerebral Aβ immunotherapy approach was used to investigate whether reducing AD pathology could restore the synaptic levels in 3xTg‐AD mice. A previous study from our group has shown that intrahippocampal anti‐Aβ injection reduces Aβ levels (Caccamo, Maldonado, Bokov, Majumder & Oddo, 2010). Here, 2 μg of 6E10 antibody was injected into the right hippocampus of 7‐month‐old 3xTg‐AD mice, and the contralateral injected hippocampus was used as an internal control. At 3 days postinjection, Aβ levels were significantly reduced (including, Aβ_38_, Aβ_40_, and Aβ_42_) in the ipsilateral hippocampi compared to the contralateral uninjected hippocampi (Figure S6A). We also measured tau levels, and no differences were observed between groups (Figure S6B,C). In an interesting manner, reduced Aβ levels led to a significant increase in multiple synaptic markers (including synaptophysin, PSD‐95, and pGluA1) as measured by western blot (Figure 5k,l).

To further demonstrate that the early synaptic alterations observed are associated with Aβ, we investigated whether dendritic spines were affected in the dentate gyrus of the hippocampus, which does not contain Aβ or tau pathology in 7‐ to 8‐month‐old 3xTg‐AD mice (Figure S7A). In particular, quantification in this region showed no differences in dendritic spine density between Ntg and 3xTg‐AD mice (Figure S7B,C). Taken together, these data indicate that Aβ accumulation is intimately related to the early synaptic deficits observed in our AD model.

## 3 DISCUSSION

Synaptic impairment is an early and critical event in the pathophysiology of AD, which better correlate with the cognitive deficits observed in patients with AD than Aβ plaques, neurofibrillary tangles, or neuronal loss (Penzes et al., 2011; Selkoe, 2002; Walsh & Selkoe, 2004). However, the mechanisms responsible for the early synaptic deficits in AD are poorly understood. This is a critical need to address, as the identification of earlier molecular mechanisms could allow for the development of more effective therapeutic approaches or treatments for AD. Here, we have identified changes in AMPAR signaling and cytoskeleton integrity that may lead to the initial synaptic defects observed in an AD mouse model. More important, the robust synaptic deficits were restored via Aβ immunotherapy, suggesting that early treatment could effectively combat the cognitive symptoms of this devastating disease.

The 3xTg‐AD mouse model is one of the most relevant animal models of AD, and these mice display cognitive and synaptic deficits at young ages, even before the appearance of plaques and tangles (Caccamo et al., 2010; Clark et al., 2015; Oddo et al., 2003). Herein, we demonstrate that synaptic function is affected early as measured by FASS‐LTP. In particular, our novel findings demonstrate that the insertion of hippocampal GluA1‐containing AMPARs during cLTP stimulation is altered in 3xTg‐AD mice. Moreover, direct electrophysiological recording of AMPAR complexes by MSM also shows that synaptic AMPAR function is significantly reduced in 3xTg‐AD mice at 7–8 months of age. Our study suggests that impairments in AMPAR activity are largely mediated by changes in AMPAR trafficking, due to reductions in multiple phosphorylation states of AMPAR subunits. The reduction in this post‐translational modification affects the incorporation of AMPARs into synapses, contributing to the associated functional deficits (Snyder et al., 2005). Taken together, our data demonstrate that synaptic AMPAR signaling is affected, leading to the early synaptic and cognitive deficits observed in this model.

The majority of excitatory synaptic processes occurs in small protrusions called dendritic spines, which are major postsynaptic sites of information processing in the brain (Carlisle & Kennedy, 2005; Fortin, Srivastava & Soderling, 2012; Parajuli et al., 2016). Synaptic function is intimately related to the structure and the number of dendritic spines, with emerging evidence suggesting that changes in these parameters are associated with the symptomatology of many different neurological disorders (Carlisle & Kennedy, 2005; Fortin et al., 2012). Our study revealed significant reductions in the number of dendritic spines in the hippocampus of 3xTg‐AD mice, suggesting that these mice have early defects in synaptic processing and neuronal communication in a brain region that is critical for learning and memory. Similar to our findings, other AD transgenic models also show reduced numbers of dendritic spines at early ages in the hippocampus, although the mechanism responsible for these dendritic spine alterations remains elusive (Chakroborty et al., 2012; Clark et al., 2015; Du et al., 2010; Jacobsen et al., 2006; Mueller et al., 2010). In addition, we also found that 3xTg‐AD mice display an increase in long immature‐type dendritic spines. These ultrastructural alterations are significant because many studies have documented that the volume of the spine head and the size of the neck are proportional to the number of postsynaptic receptors and synapse strength (Carlisle & Kennedy, 2005; Fortin et al., 2012). Therefore, our study implicates these ultrastructural changes with the early synaptic deficits observed in the 3xTg‐AD model.

The structure and function of dendritic spines are dynamically regulated by cellular pathways involving mainly the actin cytoskeleton (Cingolani & Goda, 2008; Hotulainen & Hoogenraad, 2010; Mack, Kreis & Eickholt, 2016). The actin cytoskeleton plays a pivotal role in modulating the synaptic function associated with learning and memory processes, including organization of the postsynaptic density, anchoring of postsynaptic receptors, facilitation of the trafficking of synaptic cargo, and translocalization of the translational machinery to synapses (Hotulainen & Hoogenraad, 2010). Therefore, impairments in signaling pathways that regulate actin dynamics likely account for the synaptic impairments and dendritic spine defects observed in this model. Our study demonstrates the novel findings that several actin‐related proteins, such as drebrin, spinophilin, and cortactin, are significantly reduced at early stages in 3xTg‐AD mice. These findings suggest that alterations in the mechanisms controlling the actin cytoskeleton are associated with the remarkable synaptic changes observed in these mice. Our findings are in line with previous studies indicating that drebrin, cortactin, and other actin regulators are affected in AD (Arsenault et al., 2013; Mota et al., 2014; Zhao et al., 2006). Drebrin is abundant within dendritic spines because it is required for actin clustering and the synaptic targeting of PSD95, and the suppression of drebrin expression results in a decrease in spine density (Takahashi, Mizui & Shirao, 2006). Furthermore, several studies have reported that drebrin accumulation within spines depends on AMPAR activity (Kojima & Shirao, 2007; Takahashi, Yamazaki, Hanamura, Sekino & Shirao, 2009). Therefore, the reduced AMPAR activity observed in 3xTg‐AD mice could lead to drebrin loss in the postsynaptic sites in this animal model, thereby contributing to the observed dendritic spine changes. Drebrin is also regulated by p21‐activated kinase (PAK), and several studies have shown that PAK is also reduced in AD animal models, as well as in human AD brains (Arsenault et al., 2013; Kojima & Shirao, 2007; Zhao et al., 2006). In addition, overexpression of active PAK prevents the drebrin loss induced by soluble Aβ_1‐42_ oligomers, whereas pharmacological PAK inhibition in 3xTg‐AD mice leads to drebrin loss as well as memory impairments in adult mice (Arsenault et al., 2013; Zhao et al., 2006). Our current study also demonstrates a relationship between AMPAR activity and PAK levels, which are in agreement with these previous findings. Likewise, it has been reported that cortactin, another major protein that promotes actin polymerization, is significantly downregulated in 3xTg‐AD mice (Mota et al., 2014). The reduction in cortactin levels was associated with decreased levels of the Tyr 1492‐phosphorylated NR2B subunit of the NMDARs and reduced Src kinase activity (Mota et al., 2014). These previous findings support our study demonstrating significant decreases in the phosphorylation state of multiple NMDARs subunits and cortactin. Altogether, the data suggest that signaling pathways linking NMDARs, Src kinase, and cortactin play an important role in the actin cytoskeleton stabilization and that disruption of this pathway may lead to important structural and functional synaptic deficits in young 3xTg‐AD mice.

Although plaques and tangles have traditionally been regarded as the main histopathological hallmarks of AD, a large body of evidence accumulated over the past decade suggests that soluble forms of these proteins are sufficient to trigger the development of the neurotoxicity process in AD (Berger et al., 2007; Bittner et al., 2010; Chabrier, Cheng, Castello, Green & LaFerla, 2014; Forner et al., 2017; Guerrero‐Munoz, Gerson & Castillo‐Carranza, 2015; Muller‐Schiffmann et al., 2016; Shankar et al., 2008; Spires‐Jones & Hyman, 2014; Tu, Okamoto, Lipton & Xu, 2014). These studies have clearly shown that soluble Aβ and tau oligomers are toxic to synapses and that the accumulation of these soluble isoforms correlates significantly with synaptic and memory impairments (Berger et al., 2007; Bittner et al., 2010; Chabrier et al., 2014; Forner et al., 2017; Guerrero‐Munoz et al., 2015; Muller‐Schiffmann et al., 2016; Shankar et al., 2008; Spires‐Jones & Hyman, 2014; Tu et al., 2014). In addition, we have also demonstrated that Aβ oligomers modulate the development of tau pathology and accelerate cognitive and synaptic impairments (Chabrier et al., 2014). In agreement with these previous reports, the current study shows that the synaptic deficits observed in 3xTg‐AD mice at this early stage of the disease occur before the accumulation of plaques and tangles, suggesting that soluble oligomeric Aβ and phospho‐tau isoforms can induce profound synaptic deficits in 3xTg‐AD mice by 7‐8 months of age. Moreover, several studies have shown that the CTFs of APP are also important contributing factors leading to severe synaptic and memory deficits (Lauritzen et al., 2012). Similar to these studies, we found a profound increase in CTFs levels in hippocampal synaptosome samples from 7‐ to 8‐month‐old 3xTg‐AD mice, suggesting that the CTFs may have an important role in the synaptic impairments observed at this early age. It is also crucial to note that 3‐month‐old 3xTg‐AD mice, in which Aβ and tau pathology are not present, do not show synaptic deficits. These results indicate that early synaptic impairments occur in parallel and closely related to the appearance of Aβ, CTFs, and tau‐soluble forms in this AD model. Most important, we demonstrated that early therapeutic intervention, using an anti‐Aβ immunotherapy approach, mitigates the synaptic deficits (including AMPA signaling and cytoskeletal alterations), reinforcing the idea that existing therapies, if administered early enough, could reverse the effects of AD pathology. Our study is in alignment with two new promising Aβ immunotherapy prevention/early treatment trials called the Dominantly Inherited Alzheimer Network (DIAN) and anti‐amyloid treatment for asymptomatic Alzheimer's disease (A4). Both trials have been initiated with the hope that an earlier treatment strategy might prevent downstream detrimental events in AD patients (Lemere, 2013; McDade & Bateman, 2017). In an interesting manner, our study shows that the single acute anti‐Aβ immunotherapy treatment did not change tau pathology, as previously described in a previous study from our laboratory (Oddo, Billings, Kesslak, Cribbs & LaFerla, 2004), which suggest that longer treatment is necessary to observe changes in tau.

Nevertheless, these significant findings provide important evidence of the underlying mechanisms that cause spine degeneration early in AD, which can lead to the development of new approaches to diagnose and treat AD at earlier stages. It is important to consider the current limitations of existing animal models, including the 3xTg‐AD model used in our study, which only mimic certain aspects of the full disease (LaFerla & Green, 2012). There are likely additional detrimental processes taking place early in the progression of the disease that critically affect synaptic and cognitive processes. Thus, our study and others highlight the idea that a deeper analysis into the underlying mechanisms that drive AD at early stages is necessary to develop more successful therapeutic approaches.

In summary, we report that perturbed AMPA signaling and actin cytoskeleton dysregulation occur early in the 3xTg‐AD mouse model of AD. These alterations lead to profound changes in the number and the structure of dendritic spines. AMPA dysfunction and spine alteration correlate with the presence of soluble but not insoluble Aβ and tau species, suggesting that soluble oligomeric isoforms (including Aβ and tau) are highly potent synaptotoxic molecules. In an important way, these deficits are prevented by Aβ immunotherapy, suggesting that preventive and/or earlier treatment could effectively combat synaptic dysfunction. The identification of the cellular and molecular mechanisms that impair synaptic and cognitive function during earlier stages of the disease is critical because it will increase the likelihood of developing effective therapies to stop further synaptic damage before it becomes irreversible in patients with AD.

## 4 EXPERIMENTAL PROCEDURES

### 4.1 Transgenic mice and methods

Male homozygous 3xTg‐AD and nontransgenic (Ntg) mice aged 3 and 7–8 months were used in the current study. Only male mice were used in the current study to avoid the effect of the estrous cycle on plasticity changes of hippocampal AMPA receptors (Foy, Baudry, Diaz Brinton & Thompson, 2008) that may confound our functional and expression measurements of these receptors. All mice had the same genetic background (hybrid 129/C57BL6 background). The characterization of the 3xTg‐AD mice has been described previously (Oddo et al., 2003). In brief, two independent transgenes encoding human APP_Swe_ and the human tau_P301L_ (both under the control of the mouse Thy1.2 regulatory element) were co‐microinjected into single‐cell embryos harvested from homozygous mutant PS1_M146V_ knockin (PS1‐KI) mice (Oddo et al., 2003). All animal procedures were performed in accordance with National Institutes of Health and University of California guidelines and Use Committee at the University of California, Irvine.

A detailed description of the methods including tissue processing, culture of cells, immunotherapy treatment, and electrophysiological (fluorescence analysis of single‐synapse long‐term potentiation and microtransplantation of synaptic membranes), histological (immunohistochemistry, Golgi stain, electron microscopic preparation, cresyl violet stain), and biochemical (immunoblot, dot blot, ELISA, gene expression analysis) techniques used in the current manuscript is added as supplemental data (Baglietto‐Vargas et al., 2015; Caccamo et al., 2010; Limon et al., 2012; Oddo et al., 2003; Prieto et al., 2015; Sanchez‐Varo et al., 2012).

### 4.2 Quantitative and statistical analyses

The biochemical data were quantitatively analyzed using ImageJ 1.36b software. For comparison between two groups, Student's *t* test was used, and for multiple comparisons among more than two groups, one‐way analysis of variance (ANOVA), followed by Bonferroni's comparisons, was applied using GraphPad Prism 5^®^ software (Graphpad Prism Inc., San Diego, CA, USA). The significance was set at 95% of confidence. All values are presented as mean ± *SEM*.

## AUTHOR'S CONTRIBUTION

DBV and FML conceived and designed the experiments. DBV, GAP, AL, SF, CRO, KI, LTE, JCD, and ACM performed the experiments. DBV, GAP, AL, and CRO analyzed the data. DBV, GAP, AL, SF, CRO, RA, RM, LTE, ACM, MK, JCD, CC, AG, and FML contributed to the writing of the manuscript.

## CONFLICT OF INTEREST

No potential conflict of interests relevant to this article were reported.
