# Supplementary figures and images for: Impaired AMPA signaling and cytoskeletal alterations induce early synaptic dysfunction in a mouse model of Alzheimer's disease
Source: Aging Cell. 2018 Jun 6;17(4):e12791. doi: 10.1111/acel.12791 (PMC6052400; doi:10.1111/acel.12791)

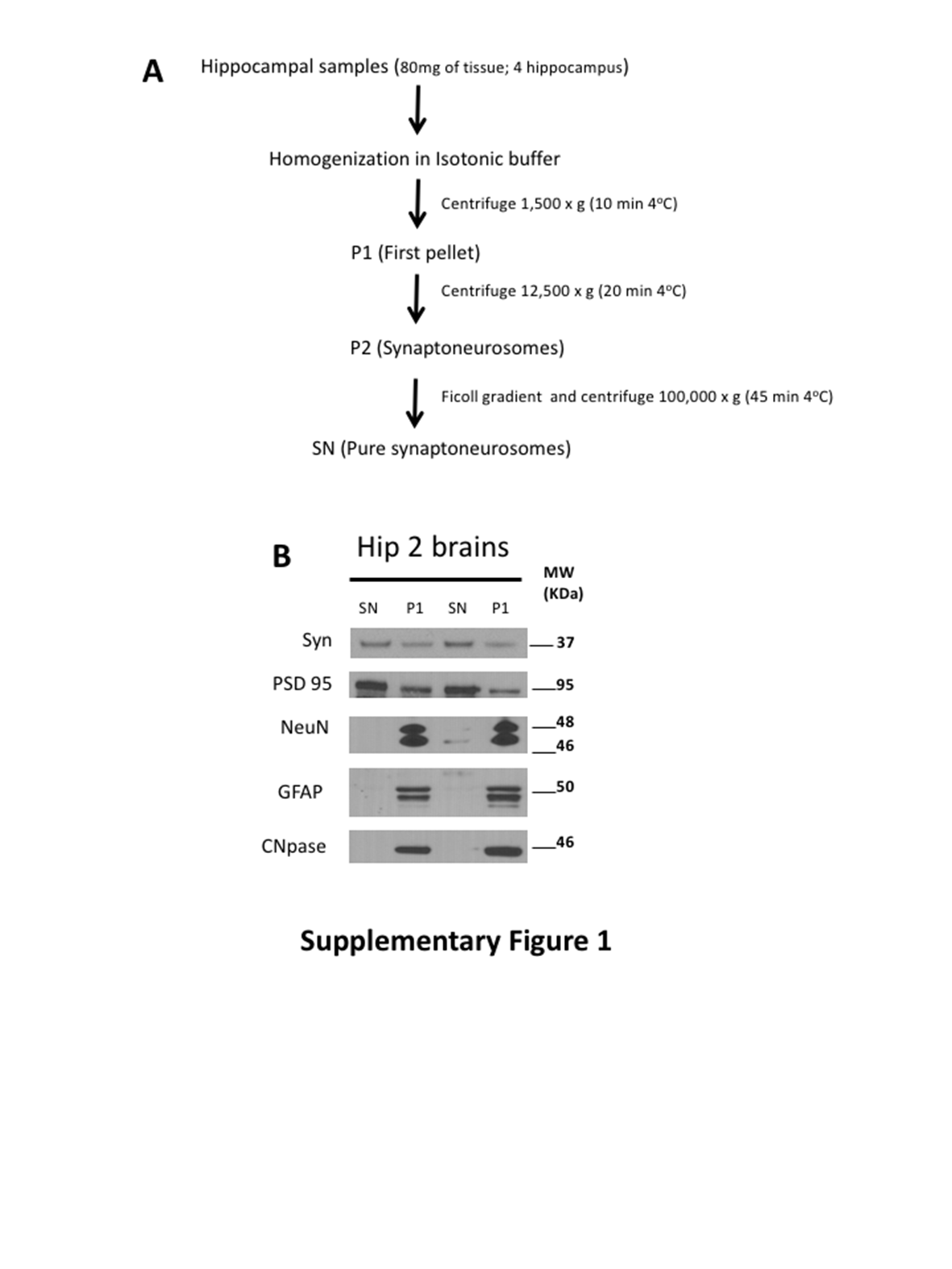

Supplement: Supplementary file 1 [file ACEL-17-na-s001.tif]

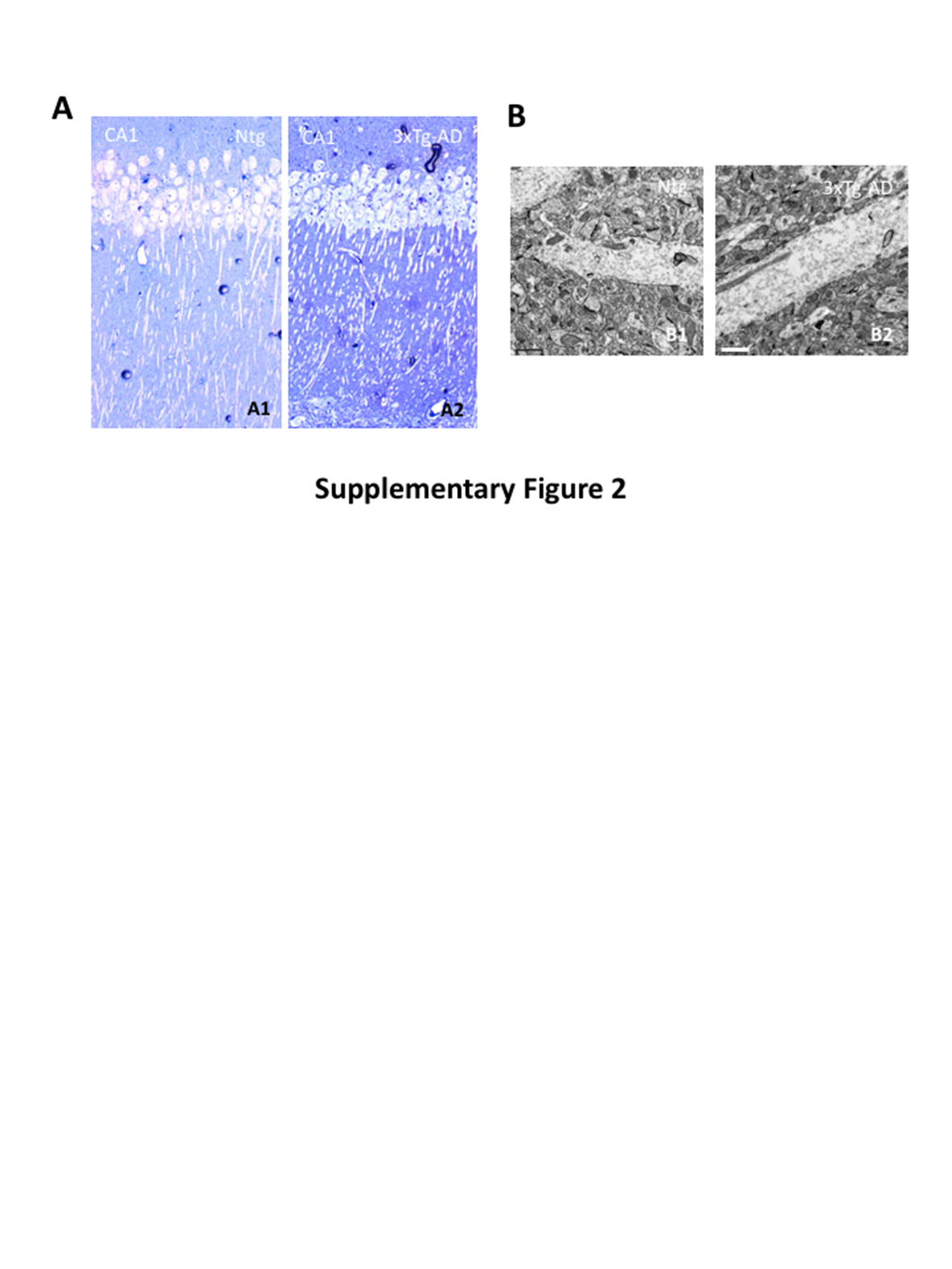

Supplement: Supplementary file 2 [file ACEL-17-na-s002.tif]

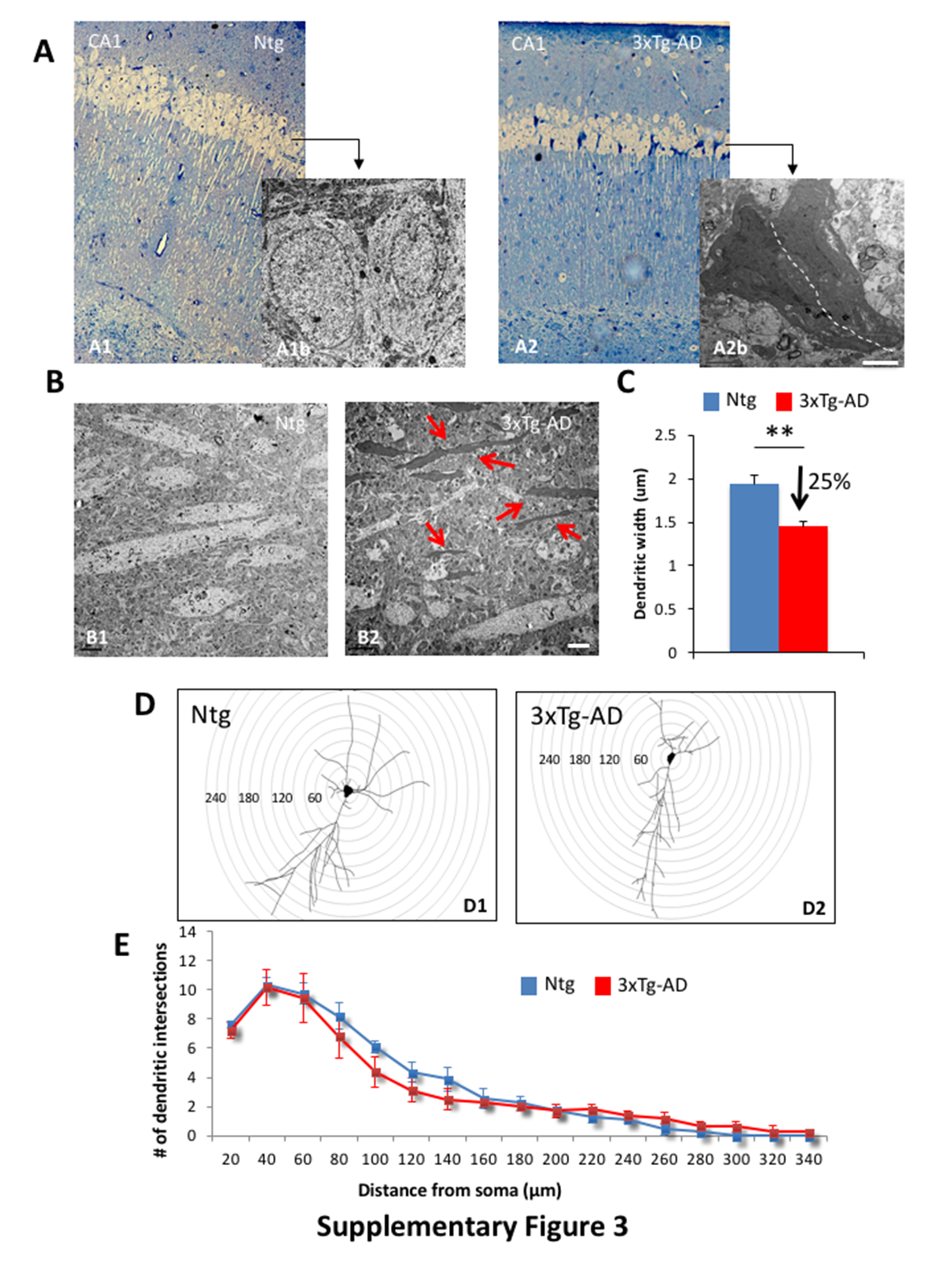

Supplement: Supplementary file 3 [file ACEL-17-na-s003.tif]

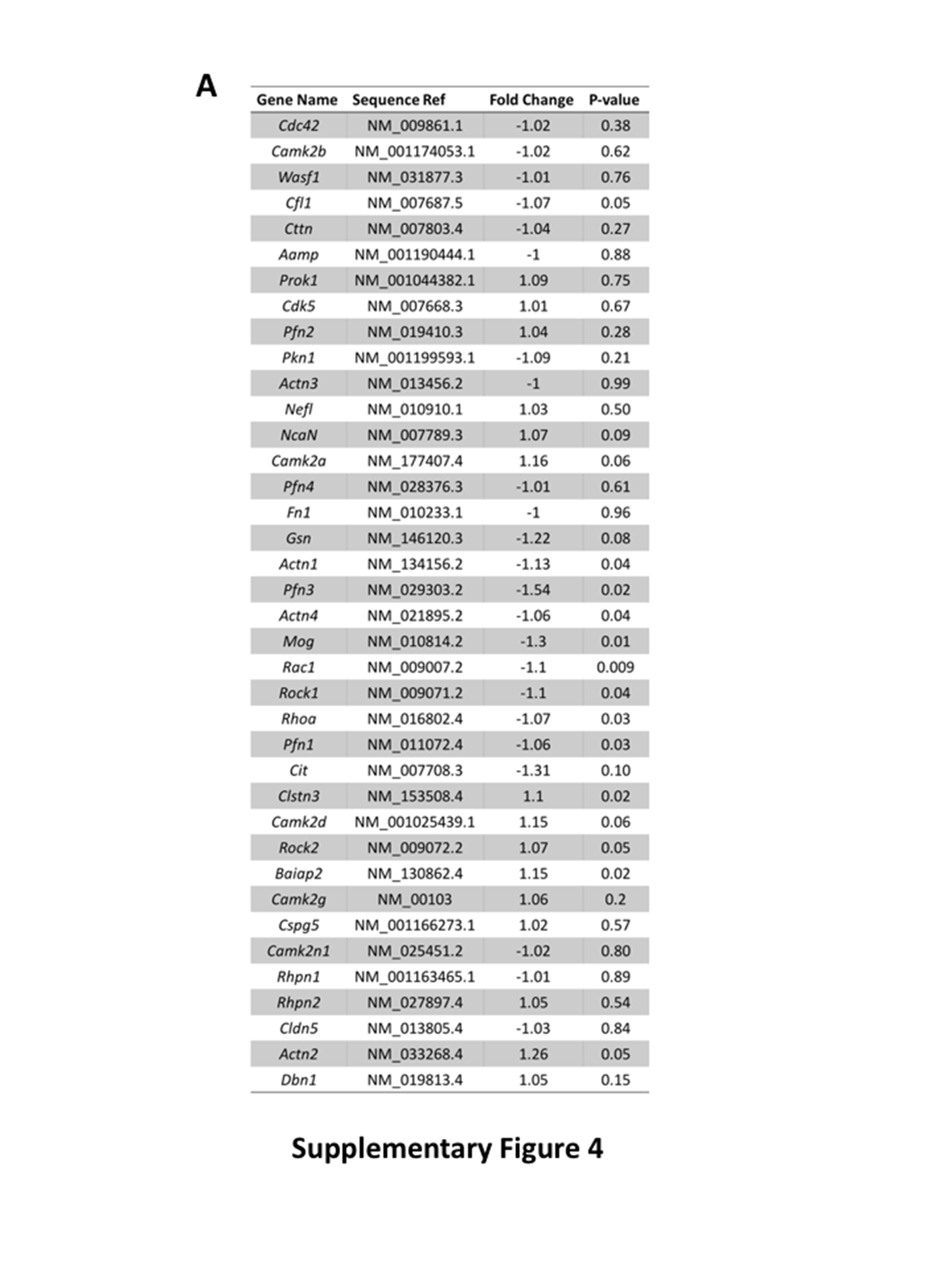

Supplement: Supplementary file 4 [file ACEL-17-na-s004.tif]

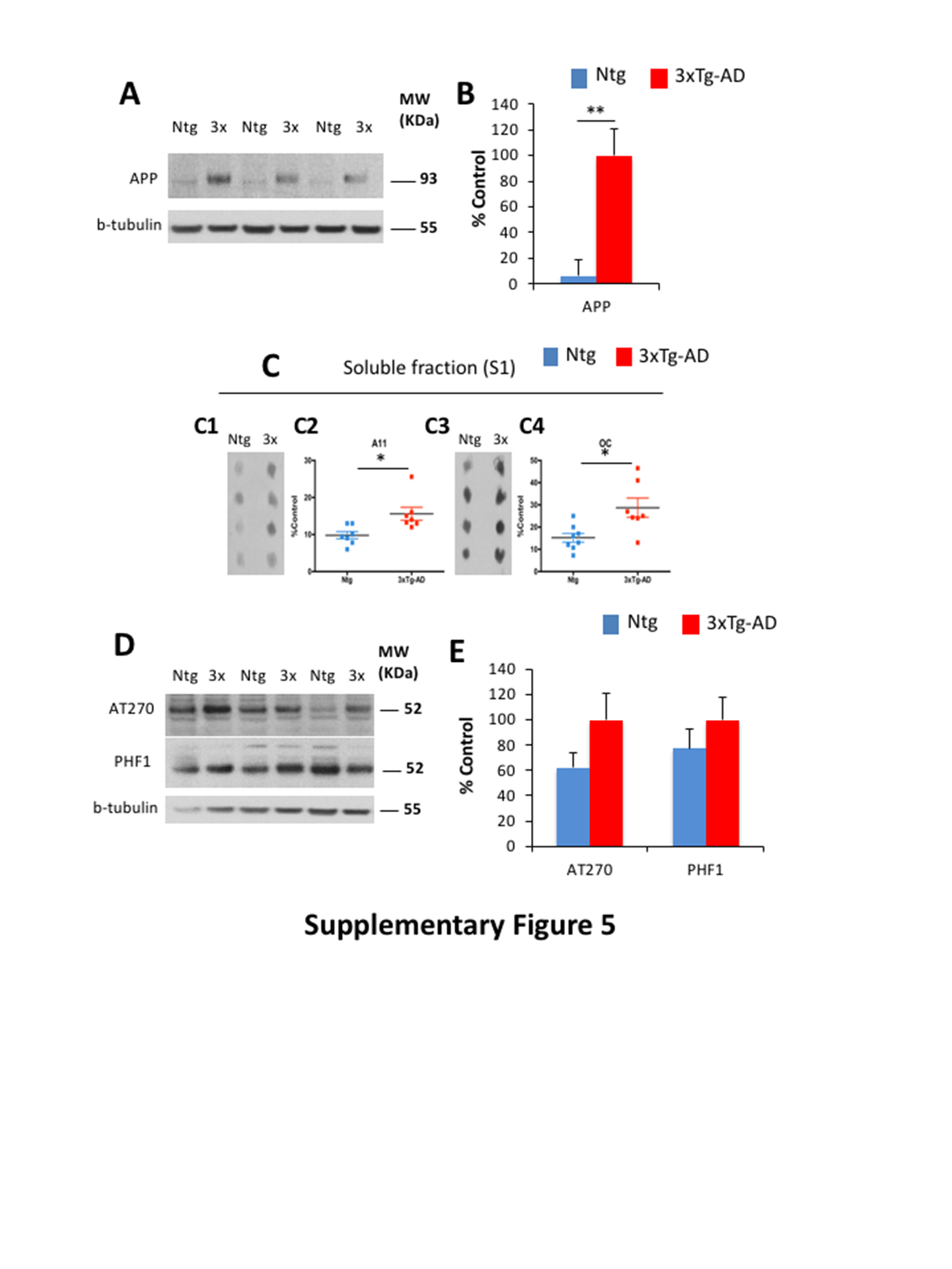

Supplement: Supplementary file 5 [file ACEL-17-na-s005.tif]

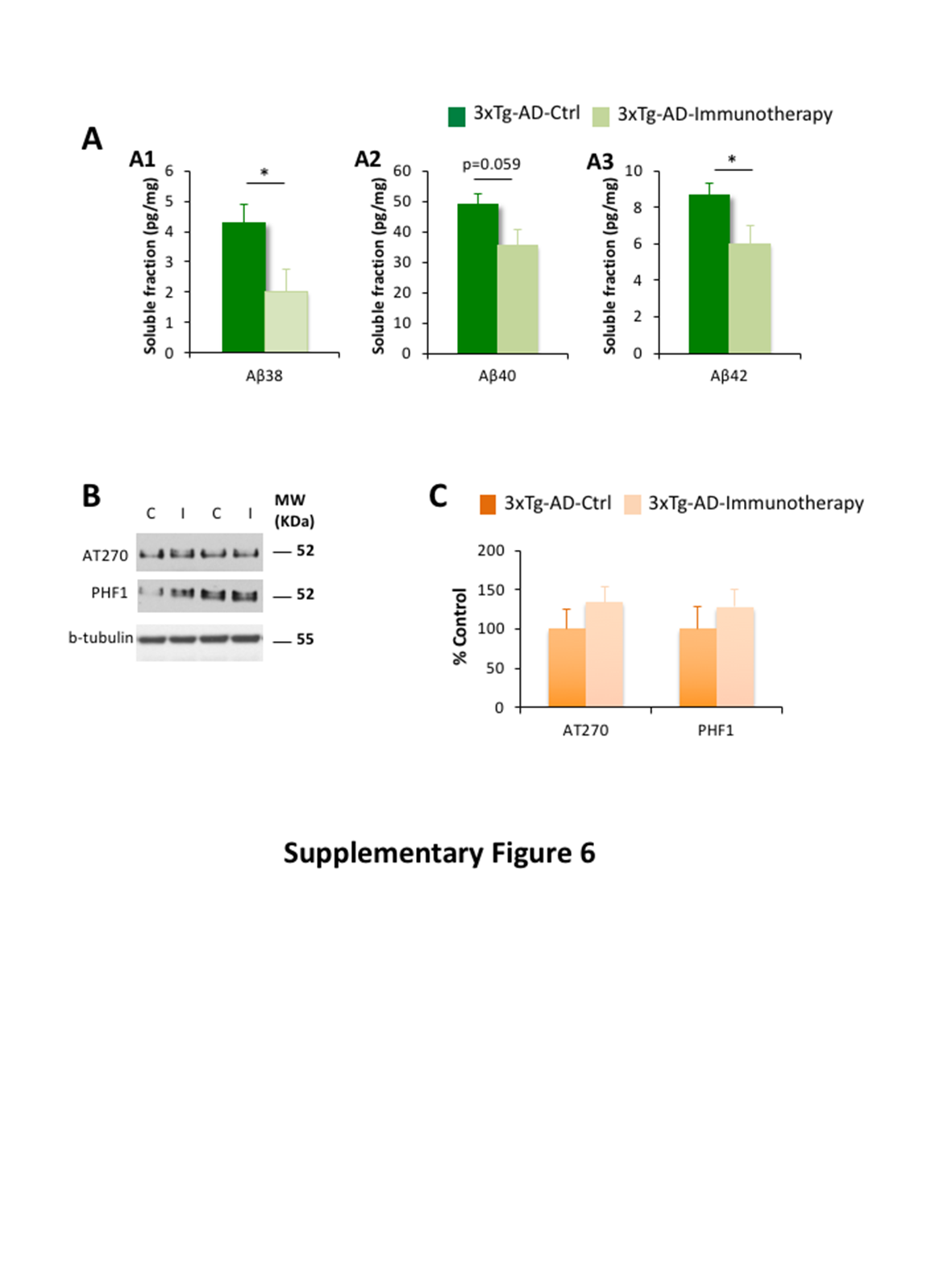

Supplement: Supplementary file 6 [file ACEL-17-na-s006.tif]

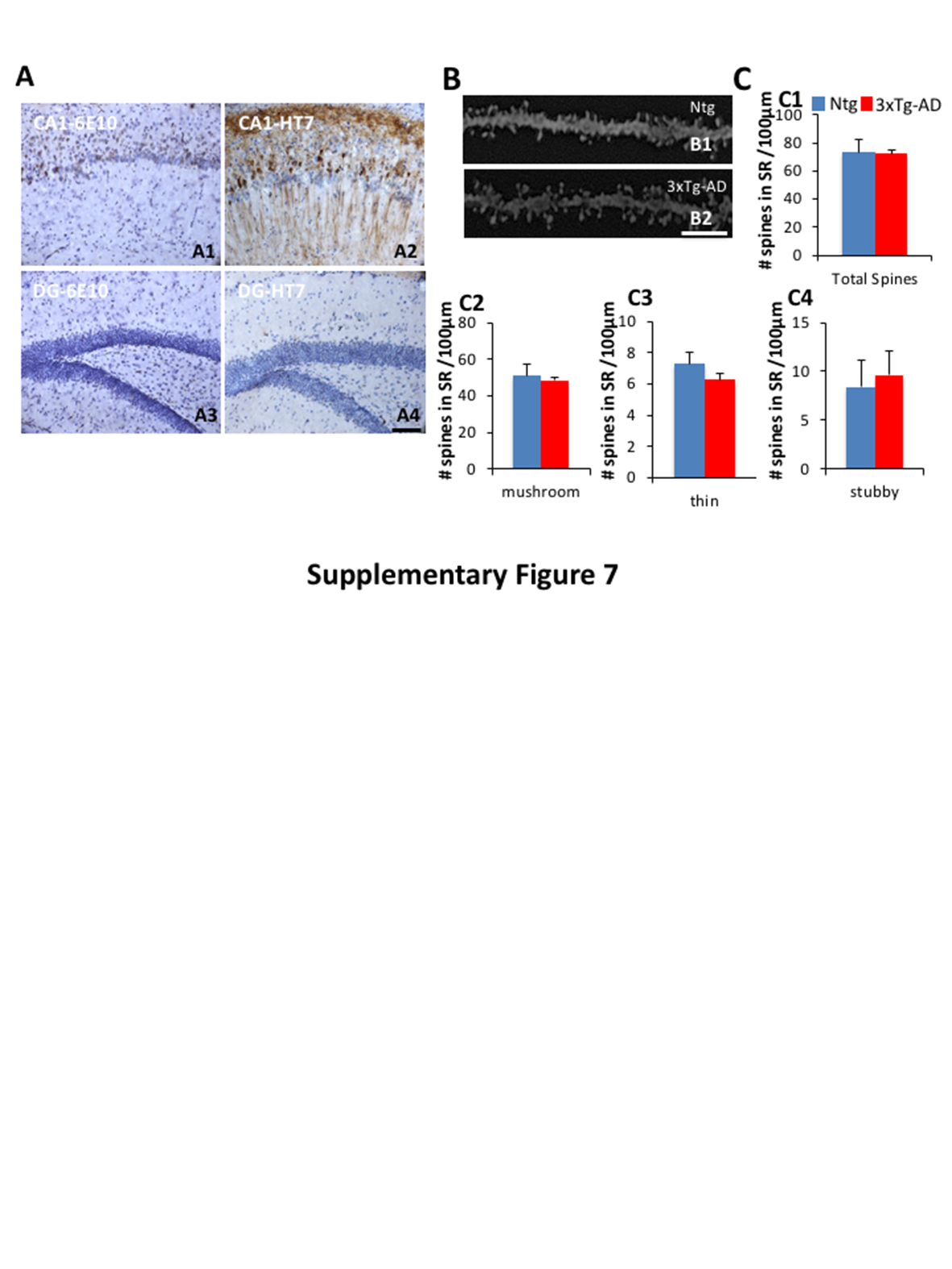

Supplement: Supplementary file 7 [file ACEL-17-na-s007.tif]
