# Supplementary material for: Impaired AMPA signaling and cytoskeletal alterations induce early synaptic dysfunction in a mouse model of Alzheimer's disease
Source: Aging Cell. 2018 Jun 6;17(4):e12791. doi: 10.1111/acel.12791 (PMC6052400; doi:10.1111/acel.12791)
Supplement: Supplementary file 8 [file ACEL-17-na-s008.docx]

| Antibody | Dilution | Method | Reference |
| --- | --- | --- | --- |
| Amyloid beta (6E10) | 1:500 | WB/IHC | SIG-39320; Covance, Emeryville, CA, USA |
| Amyloid Precursor Protein | 1:1000 | WB | 171610; EMD Millipore, Billerica, MA, USA |
| BACE-1 | 1:1000 | WB | 195111; EMD Millipore, Billerica, MA, USA |
| CNPase | 1:200 | WB | SMI-91R; Covance, Emeryville, CA, USA |
| Cofilin | 1:1000 | WB | 3312; Cell signaling, Danvers, MA, USA |
| pCofilin (Ser3) | 1:1000 | WB | 3313; Cell signaling, Danvers, MA, USA |
| Cortactin | 1:1000 | WB | 3502; Cell signaling, Danvers, MA, USA |
| pCortactin (Tyr421) | 1:1000 | WB | 4569; Cell signaling, Danvers, MA, USA |
| Cypin | 1:1000 | WB | SC-292545; Santa Cruz Biotechnology, Santa Cruz, CA, USA |
| Drebrin | 1:1000 | WB | Enzo Life Sciences, Farmingdale, NY, USA |
| GFAP | 1:2000 | WB | MAB360; EMD Millipore, Billerica, MA, USA |
| GluR1 | 1:1000 | WB | PC246; EMD Millipore, Billerica, MA, USA |
| pGluA1 (Ser831) | 1:1000 | WB | 04-823; EMD Millipore, Billerica, MA, USA |
| pGluA1 (Ser845) | 1:1000 | WB | 04-1073; EMD Millipore, Billerica, MA, USA |
| GluR2 | 1:1000 | WB | 5306; Cell signaling, Danvers, MA, USA |
| pGluA2 (Ser880) | 1:1000 | WB | 07-294; EMD Millipore, Billerica, MA, USA |
| pGluA2 (Tyr869/873/876) | 1:1000 | WB | AB2297; EMD Millipore, Billerica, MA, USA |
| NeuN | 1:1000 | WB | MAB377; EMD Millipore, Billerica, MA, USA |
| NR2B | 1:500 | WB | 06-600; EMD Millipore, Billerica, MA, USA |
| pNR2B (Tyr1472) | 1:500 | WB | M2442; Sigma-Aldrich, St. Louis, MO, USA |
| PAK1/2/3 | 1:1000 | WB | 2604; Cell signaling, Danvers, MA, USA |
| pPAK1/2/3 | 1:1000 | WB | 2605; Cell signaling, Danvers, MA, USA |
| PSD95 | 1:1000 | WB | Ab12093; Abcam, Cambridge, MA, USA |
| Spinophilin/Neurabin-II | 1:1000 | WB | 06-852; EMD Millipore, Billerica, MA, USA |
| Synaptophysin | 1:2000 | WB | Ab14692; Abcam, Cambridge, MA, USA |
| β-Tubulin | 1:1000 | WB | 2146; Cell signaling, Danvers, MA, USA |
| Tau (HT7) | 1:1000 | WB/IHC | Thermo Fisher Scientific, Rockford, IL, USA |
| pTau (Thr181-AT270) | 1:1000 | WB | Thermo Fisher Scientific, Rockford, IL, USA |
| pTau (Ser396/404-PHF1) | 1:1000 | WB | Dr. Peter Davies, Albert Einstein College of Medicine, Manhasset, NY, USA |
