# Supplementary material for: Impaired AMPA signaling and cytoskeletal alterations induce early synaptic dysfunction in a mouse model of Alzheimer's disease
Source: Aging Cell. 2018 Jun 6;17(4):e12791. doi: 10.1111/acel.12791 (PMC6052400; doi:10.1111/acel.12791)
Supplement: Supplementary file 9 [file ACEL-17-na-s009.docx]

**Supplementary Experimental Procedures**

***Tissue preparation***

After deep anesthesia with sodium pentobarbital (60 mg/Kg), Ntg and 3xTg-AD mice were perfused transcardially with 0.1M phosphate-buffered saline (PBS, pH 7.4). Next, brain tissue was used to collect synaptosomes ([Sanchez-Varo *et al.* 2012](#_ENREF_40)), to stain with Golgi solution ([Baglietto-Vargas *et al.* 2015](#_ENREF_2)), for electron microscopic analysis ([Sanchez-Varo *et al.* 2012](#_ENREF_40)) or for immunohistological staining.

***Synaptosome extracts***

Synaptosome extracts were prepared as described previously ([Sanchez-Varo *et al.* 2012](#_ENREF_40)). Briefly, the tissue (the hippocampus from two mice) was homogenized (using a Dounce homogenizer) in 0.32 M sucrose, 10 mM Tris–HCl (pH 7.4) buffer (buffer A) containing complete protease and phosphatase inhibitor cocktails (Sigma-Aldrich, St.Louis, MO, USA). After homogenization, the crude synaptosomal fraction (synaptosomes plus mitochondria) was isolated by two sequential centrifugations (1,500 x g, 10 min followed by 12,500 x g, 20 min; at 4°C). The crude synaptosomes were resuspended in 13% (final concentration) Ficoll 400 (in buffer A) and layered on the bottom of a discontinuous gradient, composed of buffer A and 7% Ficoll (in buffer A). The gradients were centrifuged at 100,000 x g (45 min at 4°C) and the synaptosomes were isolated at the 7.5–13% interface. After washing (twice with buffer A), the protein content of the synaptosomal fractions was determined using the Bradford assay. Fresh synaptosomes were used for Fluorescence Analysis of Single-Synapse Long-Term Potentiation (FASS-LTP). For western blots and microtransplantation of Synaptic Membranes (MSM) experiments synaptosomal preparations were stored at -80^0^ C. Western-blot analysis demonstrated that the synaptosome extracts were rich in synaptic markers and that nuclear neuronal or glial markers were not present (Figure S1A).

***Immunoblotting***

Equal amounts of protein (5 μg) were separated on 10% Bis-Tris gel (Invitrogen, Carlsbad, CA), and transferred to nitrocellulose membranes. Membranes were blocked for 1 h in 5% (w/v) suspension of bovine serum albumin (BSA; Gemini Bio-Products, West Sacramento, CA, USA) in 0.2% Tween 20 Tris-buffered saline (TBS) (pH 7.5). After blocking, the membranes were incubated overnight at 4°C, with one of the following primary antibodies described in table 1. The membranes were washed in Tween 20-TBS for 20 min and incubated at 20°C with the specific secondary antibody at a dilution of 1:10000 (Pierce Biotechnology) for 60 min. The blots were developed using Super Signal (ThermoFisher Scientific, Rockford, IL, USA).

***Dot-blot***

Equal amounts of protein (3 μg) were transferred to nitrocellulose membranes. Membranes were blocked for 1 h in 5% (w/v) suspension of BSA (Gemini Bio-Products) in TBS containing 0.2% Tween 20 (pH 7.5). After blocking, the membranes were incubated overnight at 4°C, with one of the following primary antibodies: anti-A11 (1:1000, Life technologies, Grand Island, NY) and anti-OC (1:3000, EMD Millipore, Billerica, MA). The membranes were washed in Tween 20-TBS for 20 min and incubated at 20°C with the specific secondary antibody at a dilution of 1:10000 (Pierce Biotechnology) for 60 min. The blots were developed using Super Signal (Thermo Scientific).

***RNA extraction and gene expression analysis***

RNA was isolated from half hippocampi of 7 month-old Ntg and 3xTg-AD mice (n=4) using RNA Plus Universal Mini Kit (Qiagen, Hilden, Germany), and was hybridized and multiplexed with NanoString probes, according to the manufacturer’s instructions. The synaptic genes were selected for analysis and probes were designed and synthesized by NanoString nCounter TM technologies (Nanostring, Seattle, WA). Nanostring technology has implemented thorough quality control (QC) checks to ensure reliability of the data: imaging QC to discard visualization and image digitization artifacts, binding density QC to avoid image saturation, inclusion of positive controls to determine limit and linearity of the detection, and a series of negative controls to determine background. All these QC help to reduce the technical variability of the measurements. 20ng/μl of total RNA was used to measure the expression of 37 cell adhesion and cytoskeleton synaptic genes (*Actn1, Actn2, Actn3, Actn4, Dbn1, Cfl1, Camk2a, Camk2b, Camk2g, Camk2n1, Rac1, Cdc42, Rhoa, Rock1, Rock2, Pfn1, Pfn2, Pfn3, Pfn4, Cit, Pkn1, Prok1, Rhpn1, Rhpn2, Gsn, Cttn, Wasf1, Cdk5, Baiap2, Cldn5, Nefl, Fn1, Aamp, Cspg5, Clstn3, Mog* and *Ncan*) genes. Counts for target genes were normalized to housekeeping genes (*Alas, Eef1g, G6pdx, Gapdh, Gusb, Hprt, Oaz1, Polr1b, Polr2a, Ppia, Rpl19, Sdha, Tbp* and *Tubb5*) to account for variability in the RNA content. The background signal was calculated as the mean value of the negative hybridization control probes. We used nSolver 4.0, which is an analysis software that uses all QC information, in conjunction with the housekeeping genes, to calculate fold changes and perform the statistic tests. Gene expression values were presented as fold change with respect to the Ntg group and P values were calculated using a t-test with nSolver 4.0 software.

***Fluorescence analysis of single-synapse long-term potentiation (FASS-LTP)***

Fresh hippocampal synaptosome P2 fractions were obtained from Ntg and 3xTg-AD mice and stimulated using a chemical long-term potentiation approach (cLTP), as previously described ([Prieto *et al.* 2015](#_ENREF_39)).

***Microtransplantation of synaptic membranes (MSM)***

MSM was done as described previously ([Limon *et al.* 2012](#_ENREF_26)). Aliquotes of synaptosomes (P2 fraction) from Ntg and 3xTg-AD hippocampi used for western blots were sonicated (3 x 5 sec, 1 min interval between sonications) to form proteoliposomes able to fuse with the membrane of injected *Xenopus* oocytes. Fifty nL of synaptic membranes (2 mg/mL protein concentration) were injected into stage V-VI *Xenopus* oocytes and, from 18 to 36 h after injection, membrane currents were recorded from oocytes voltage clamped at −80 mV. *s*-AMPA and cyclothiazide (CTZ) were bought from Tocris (Tocris, Minneapolis, MN, USA).

***Golgi staining***

Ntg and 3xTg-AD mice were perfused transcardially with 0.1M phosphate-buffered saline (PBS, pH 7.4) and their brains were processed using a superGolgi Kit (Bioenno Tech LLC, Santa Ana, CA), as described previously ([Baglietto-Vargas *et al.* 2015](#_ENREF_2)).

***Dendritic and spine analysis***

Stereological quantifications were performed using Neurolucida software (MBF Bioscience, Williston, VT, USA) to determine the number of spines in the stratum radiatum (sr) of the hippocampal CA1 and dentate gyrus (dg) region, respectively. Briefly, every second section was used through the entire antero-posterior extent of the hippocampus (between -1.46 mm anterior and -3.40 mm posterior to Bregma according to the atlas of Franklin and Paxinos, Third Edition, 2007). The sr in CA1 and dg region was defined using a 5x objective and spines were counted using a 100x/1.4 objective. The coefficient of error (CE) value for each individual animal ranged between 0.03 and 0.08. Dendritic spine length was traced using a 100x/1.4 objective and data were analysed via Neurolucida Explorer software. For dendritic morphological analysis, 5 neurons per animal (n=6) in CA1 hippocampal area were traced using Neurolucida software and evaluated using Sholl analysis. Dendritic width was measured using Image J software in electron microscopic images (10 images per animal for a total of 5 mice per group).

***Electron microscopic preparation***

Electronic microscopy was performed as described in detail previously ([Sanchez-Varo *et al.* 2012](#_ENREF_40)). Ntg and 3xTg-AD mice were perfused transcardially with 0.1 M phosphate buffered saline (PBS)/1% heparin, pH 7.4, followed by 2.5% glutaraldehyde–2% paraformaldehyde in 0.1 M phosphate buffer (PB), pH 7.4. After being removed, the brains were post-fixed in the same fixative overnight at 4°C, washed several times with PB, sectioned at 50 or 100 µm thickness in the coronal plane on a vibratome (Leica VT1000M) and serially collected in wells containing cold PB and 0.02% sodium azide. Then 100-µm sections were fixed in 2% osmium tetroxide in 0.1 M PB and dehydrated, to be finally embedded in Araldite (EMS, USA). Tissue blocks were cut serially into semithin (1.5 µm) with a diamond knife in a Leica ultramicrotome (EM UC6), placed on slides, stained with 1% toluidine blue and explored with the light micro- scope for amyloid plaques. Next, selected areas from semithins were cut in ultrathin sections. Ultrathin sections were placed on Formvar-coated grids and stained with uranyl acetate and lead citrate before being examined with an electron microscope (FEI Tecnai Spirit, OR, USA).

***Immunohistochemistry***

Coronal free-floating sections (40μm thick) were pretreated with 3% H2O2/3% methanol in Tris-buffered saline (TBS) for 30 min to block endogenous peroxide activity. After TBS wash, sections were incubated first in TBS with 0.1% Trition X-100 (TBST) for 15 min, and then in TBST with 2% bovine serum albumin (BSA, Sigma-Aldrich) for 30 min. Sections were incubated with anti-6E10 (1:1000; Covance Research Products, Denver, PA, USA) and HT7 (1:1000; ThermoFisher Scientific) in TBS + 5% normal horse serum overnight at 4^0^C. Sections were then incubated with biotinylated anti-mouse, 1:500 in TBS + 2%BSA + 5% normal serum for 1hr at 20^0^C, followed by Vector ABC kit and DAB reagents (Vector Laboratories, Burlingame, CA, USA) to visualize staining. Next, the sections were co-staining with cresyl violet stain.

For fluorescence staining, sections were incubated in secondary donkey anti-mouse Alexa-fluor 488 for the 6E10 antibody and donkey anti-mouse Alexa-fluor 555 for the HT7 antibody. In addition, sections were stained with DAPI. The images were collected with Microlucida software and using a 20x and 40x objective with an AxioImager M2 with a 64-bit PC fluorescence camera (Zeiss, Irvine, CA, USA).

***Cresyl violet staining***

Sections were mounted on slides and air-dried overnight in a dark room. Slides were re-hydrated in de-ionized H_2_0 for 15 min followed by PBS, pH 7.4, for 15 min. Slides were then incubated in 1% cresyl violet acetate solution (Merck, Whitehouse Station, NJ, USA) for 5 min following dehydration in graded ethanol’s (70%, 96% + acetic acid, and 100%) for 5 min, respectively. Finally, slides were placed in xylene for 5 min and cover-slipped using DPX (VWR) mounting medium.

***ELISA***

Aβ levels were quantiﬁed using the MSD96-well multi-spot 6E10 Aβ triple ultra-sensitive assay kit, according to the manufacture’s instruction (Meso Scale Discovery, Rockville, MD, USA) as previously described ([Baglietto-Vargas *et al.* 2015](#_ENREF_2)).

***Primary cell culture and cLTP stimulation***

Hippocampal primary cells were collected from postnatal day 0 C57BL/6J mice. Primary cells were grown in Neurobasal medium with penicillin and streptomycin and supplemented with GlutaMAX and B-27 (ThermoFisher Scientific). Primary cells were fed twice a week. 14 to 16 day-old cells were treated for 2 h with 100 nM amyloid beta-derived diffusible ligands 1-42 (ADDLs). ADDLs were prepared according to the protocol previously described by Klein ([Lacor *et al.* 2007](#_ENREF_22)). ADDLs concentration was determined by Nanodrop (ThermoFisher Scientific) immediately before applying to the cells. After ADDLs incubation, cLTP was induced using a modified protocol described by Park *et al.* (2006) ([Park *et al.* 2006](#_ENREF_37)). First, cells were washed for 5 min with extracellular solution containing [mM]: 120 NaCI, 3 KCl, 2 CaCl2, 2 MgCl2 15 glucose, 15 HEPES, pH 7.4. For glycine stimulation, cells were treated for 10 min with 0.2mM glycine in a solution containing [mM]: 150 NaCI, 5 KCl, 2CaCl2, 30 glucose, 10 HEPES, 0.001 strichnine, 0.02 bicuculline, pH 7.4. Cells were returned to extracellular solution and incubated for an additional 50 min before lysis. For collection, cells were washed with ice-cold PBS. Then, M-PER complemented with proteases and phosphatases inhibitors (ThermoFisher Scientific) was added, cells were scrapped and centrifuged at 12,000 x g for 10 min at 4°C. Protein concentration in the supernatant was determined using the Bradford assay (Biorad).

***Aβ immunotherapy***

7 month-old male 3xTg-AD mice received a single injection of 2μg of 6E10 into the right hippocampus as described previously ([Caccamo *et al.* 2010](#_ENREF_6)). The left uninjected hippocampus was used as internal control. The mice were killed 3 days after antibody delivery. Crude synaptosome (P2) extracts were prepared for western-blot analysis, and Aβ levels were measured by ELISA.

**Supplementary Figures**

***Supplemental Figure S1. Validation of synaptosome extracts.*** A) Diagram of synaptosome sample preparation. B) Western-blot analysis was used to evaluate the purity of the hippocampal synaptosome samples (SN). The results indicate that the synaptosome extracts showed elevated levels of the synaptic markers synaptophysin (Syn) and PSD-95 and a total absence of nuclear protein (recognized by the NeuN antibody), astrocytes (recognized by the GFAP antibody) and oligodendrocytes (recognized by the CNpase marker). Pellet 1 (P1) samples were positive for all markers.

***Supplemental Figure S2. Dendritic spine morphology in 3 month-old mice.*** A) Semithin slice images from Ntg (A1) and 3xTg-AD (A2) mice showed similar morphological and structural cell appearance. Detailed electron microscopic images in Ntg (B1) and 3xTg-AD (B2) mice showed no differences in dendritic spine projections and spines. Scale bars: 1 μm (B1 and B2).

***Supplemental Figure S3. Dendritic morphology and complexity in CA1 pyramidal cells.*** A) Semithin slices from Ntg (A1) and 3xTg-AD (A2) mice showed an increased number of cells that accumulate toluidine blue solution. Electron microscopic images in Ntg (A1b) and 3xTg-AD (A2b) mice showed that the cells which accumulate the colorant (toluidine blue) had an electrodense and pyknotic-like nuclear appearance. B-C) Electron microscopic images of the Ntg (B1) and 3xTg-AD (B2) stratum radiatum of the CA1 hippocampal subfield (red arrows indicate electrodense and narrow dendritic projections in 3xTg-AD mice). Quantification of B showed a significant decrease in the dendritic width in 3xTg-AD compared to Ntg mice (25±3.33, t-test p< 0.01). D) Diagram of Sholl dendrite analysis measured dendrite density by placing a series of concentric circles spaced at 20-μm intervals centered on the soma in Ntg (D1) and 3x-Tg-AD (D2). E) Quantification of D by Sholl method showed no significant differences in dendritic complexity between Ntg and 3xTg-AD mice. The values represent the mean ± SEM (n=6-7 per group). ** *p* < 0.01. Scale bars: 2 μm (A1b, A2b, B1 and B2).

***Supplemental Figure S4. Summary of gene expression in 3xTg-AD mice.*** A) Using NanoString nCounter technology, we investigated gene expression of multiple actin related genes. Several genes reported an up-regulated expression, including calsyntenin 3 (*Clstn3*) (1.1-fold, *p* < 0.05) and brain-specific angiogenesis inhibitor 1-associated protein 2 (*Baiap2*) (1.15-fold, *p* < 0.05), meanwhile most of the genes showed a significant down-regulation such as alpha-actinin-1 (*Actn1*) (-1.13-fold, *p* < 0.05), profilin-3 (*Pfn3*) (-1.54-fold, *p* < 0.05), alpha-actinin-4 (*Actn4*) (-1.06-fold, *p* < 0.05), myelin oligodendrocyte glycoprotein (*Mog*) (-1.3-fold, *p* < 0.05), Ras-related C3 botulinum toxin substrate 1 (*Rac1*) (-1.1-fold, *p* < 0.01), Rho-associated protein kinase 1 (*Rock1*) (-1.1-fold, *p* < 0.05), Ras homolog gene family member A (*Rhoa*) (-1.07-fold, *p* < 0.05) and profiling 1 (*Pfn1*) (-1.07-fold, *p* < 0.05) in Ntg mice compared to 3xTg-AD mice.

***Supplemental Figure S5. Extrasynaptic APP/Aβ and tau pathology in 3xTg-AD mice.*** A) Immunoblot analysis of Amyloid precursor protein (APP) from hippocampal S1 homogenates of 7-8 month-old Ntg and 3xTg-AD mice shown as alternating lanes. B) Quantification of APP normalized to β-tubulin and expressed as a % of control, showed only human APP expression, recognized with the 6E10 antibody, in 3xTg-AD compared to Ntg mice. C) Dot blot analysis revealed a significant increase in the level of Aβ-oligomers recognized by the antibodies A11 (41.97±15.22, * *p* < 0.05, t-test) and OC (72.89±28.90, * *p* < 0.05, t-test) in 3xTg-AD compared to Ntg mice. D) Immunoblot analysis of AT270 and PHF1 from hippocampal S1 homogenates of 7-8 month-old Ntg and 3xTg-AD mice shown as alternating lanes. E) Quantification of AT270 and PHF1 normalized to β-tubulin and expressed as a % of control, showed an increase in both markers in the 3xTg-AD. The values represent the mean ± SEM (n=7 per group). * *p* < 0.05.

***Supplemental Figure S6. Soluble Aβ levels is reduced in P2 synaptosomes of 3xTg-AD mice treated with 6E10 immunotherapy.*** A) Aβ measurements by sandwich ELISA of soluble (Aβ38: 53.58±17.34, * *p* < 0.05, t-test, Aβ40: 27.79±10.76, *p=*0.059, t-test and Aβ42: 31.39±11.93, * *p* < 0.05, t-test) fractions were significantly decreased in 3xTg-AD-6E10 compared to 3xTg-AD-Ctrl mice. B) Immunoblot analysis of AT270 and PHF1 from hippocampal S1 homogenates of 7-8 month-old Ntg and 3xTg-AD mice shown as alternating lanes. E) Quantification of AT270 and PHF1 normalized to β-tubulin and expressed as a % of control, showed no differences. The values represent the mean ± SEM (n=6 per group). * *p* < 0.05.

***Supplemental Figure S7. Dendritic spine densities are not affected in the dentate gyrus of 3xTg-AD mice.*** A) Light microscopic images of CA1 region (A1 and A2) and dentate gyrus (A3 and A4) stained with 6E10 antibody (A1 and A3) or HT7 (A2 and A4) showed significant amyloid and tau pathology in the CA1 subfield (A1 and A2), whereas no pathology was observed in the dentate gyrus region (A3 and A4). The sections were counterstained with cresyl violet. B-C) Spine quantification per dendritic length showed no differences in spine density in Ntg compared to 3xTg-AD mice (C1) in the dentate gyrus. In addition, quantification based on the type of spines (including mushroom, thin and stubby) demonstrates no differences in Ntg compared to 3xTg-AD mice (C2-C4). Scale bars: 100 μm (A1-A4) and 5 μm (B1 and B2). The values represent the mean ± SEM (n=4-7 per group).

***Table 1. List of the antibodies used for immunoblots and Immunohistochemistry.*** Here we have provided the concentration, incubation and reference of the different antibodies used for immunoblot and immunohistochemical analyses.
